# Sorting the Wheat From the Chaff: Programmed Cell Death as a Marker of Stress Tolerance in Agriculturally Important Cereals

**DOI:** 10.3389/fpls.2019.01539

**Published:** 2019-11-26

**Authors:** Alysha Chua, Laurence Fitzhenry, Cara T. Daly

**Affiliations:** Department of Science, Waterford Institute of Technology, Waterford, Ireland

**Keywords:** programmed cell death, plant stress tolerance, root hair assay, cereals, basal tolerance, induced tolerance, stress phenotypes

## Abstract

Conventional methods for screening for stress-tolerant cereal varieties rely on expensive, labour-intensive field testing and molecular biology techniques. Here, we use the root hair assay (RHA) as a rapid screening tool to identify stress-tolerant varieties at the early seedling stage. Wheat and barley seedlings had stress applied, and the response quantified in terms of programmed cell death (PCD), viability and necrosis. Heat shock experiments of seven barley varieties showed that winter and spring barley varieties could be partitioned into their two distinct seasonal groups based on their PCD susceptibility, allowing quick data-driven evaluation of their thermotolerance at an early seedling stage. In addition, evaluating the response of eight wheat varieties to heat and salt stress allowed identification of their PCD inflection points (35°C and 150 mM NaCl), where the largest differences in PCD levels arise. Using the PCD inflection points as a reference, we compared different stress effects and found that heat-susceptible wheat varieties displayed similar vulnerabilities to salt stress. Stress-induced PCD levels also facilitated the assessment of the basal, induced and cross-stress tolerance of wheat varieties using single, combined and multiple individual stress exposures by applying concurrent heat and salt stress in a time-course experiment. Two stress-susceptible varieties were found to have low constitutive resistance as illustrated by their high PCD levels in response to single and combined stress exposure. However, both varieties had a fast, adaptive response as PCD levels declined at the other time-points, showing that even with low constitutive resistance, the initial stress cue primes cross-stress tolerance adaptations for enhanced resistance even to a second, different stress type. Here, we demonstrate the RHA’s suitability for high-throughput analysis (∼4 days from germination to data collection) of multiple cereal varieties and stress treatments. We also showed the versatility of using stress-induced PCD levels to investigate the role of constitutive and adaptive resistance by exploring the temporal progression of cross-stress tolerance. Our results show that by identifying suboptimal PCD levels *in vivo* in a laboratory setting, we can preliminarily identify stress-susceptible cereal varieties and this information can guide further, more efficiently targeted, field-scale experimental testing.

## Introduction

The global population is estimated to reach 9.7 billion by 2050 (United Nations, 2017). Consequently, agriculture systems must be rooted solidly in practices that sustain and enhance our natural environments but must also evolve to meet rising food demands. Until recently, a relatively predictable climate has allowed commercial farmers to prioritise high-yielding crops over stress tolerant varieties. However, the potential gains of high-yielding varieties are redundant if plants are liable to succumb to stress as novel climate abnormalities cause crops to have more frequent encounters with unique abiotic and biotic stress combinations (Mittler, 2006). As a consequence of modified plant physiology and a weakened defence system, crop yield is negatively impacted as plants become more susceptible to pathogens and have lower competitive ability against weeds (Pandey et al., 2017). There is a growing consensus that we need to broaden the focus from production of high-yielding crops, to developing more stress-tolerant varieties as yield improvements must not come at the expense of environment and ecosystem damage (Coleman-Derr and Tringe, 2014; Meena et al., 2017).

Over the years, researchers have developed a diverse range of molecular biology techniques to investigate the different stress-response phases that underpin plant stress tolerance, such as transcriptomics (mRNA transcriptional and post-transcriptional analysis, e.g. micro-RNA and small interfering RNAs) (Chinnusamy et al., 2010), proteomics (2-dimensional liquid chromatography, polyacrylamide gel electrophoresis, difference gel electrophoresis) (Ahmad et al., 2016), metabolomics (gas/liquid chromatography-mass spectrometry, capillary electrophoresis and nuclear magnetic resonance spectroscopy) (Obata and Fernie, 2012), and phenomics (high-throughput phenotyping) (Singh et al., 2018). These high-throughput methods integrate large amounts of information to generate a high-resolution picture of the plant stress response but are often labour-intensive processes that involve significant technical expertise. In contrast, biochemical and physiological techniques are cheaper, quicker and offer useful stress biomarkers. One such biochemical marker is investigation of cellular oxidative damage. Used as a common measurement of plant stress tolerance, excessive reactive oxygen species (ROS) damage subcellular components and trigger programmed cell death (PCD) (Petrov et al., 2015). Common methods for quantifying oxidative damage include total antioxidant capacity, lipid peroxidation and measurement of non-enzymatic and enzymatic antioxidant levels (Elavarthi and Martin, 2010; Jambunathan, 2010). Other biomarkers include fluctuations in cell osmolyte levels which regulate cell volume and maintain osmotic balance during stress onset (Verslues, 2010), while ion quantification is used to screen plants for salt tolerance as the ability to partition and cycle ions through the different tissues is vital for surviving salt stress (Munns et al., 2010).

In the present work we show how PCD can be used as a quick effective tool to identify stress-tolerant cereal varieties. PCD is a normal facet of plant growth and development activated by developmental and environmental factors, but is also a protective mechanism during abiotic and biotic stress onset (Petrov et al., 2015). PCD describes a highly organised sequence of events that leads to the controlled disassembly of the cell and is characterised by the distinctive Ca^2+^-dependent retraction of the cytoplasm (Kacprzyk et al., 2017). Conversely, necrosis is associated with uncontrolled Ca^2+^-independent cell death that occurs when cells cannot withstand overwhelming cellular stress (Kacprzyk et al., 2017). Necrotic death is characterised by a loss of plasma membrane integrity, resulting in impaired osmoregulation and the cellular influx of water and ions, causing the cell to swell and rupture, releasing their cellular contents (Lockshin and Zakeri, 2004). PCD plays an important role in the plant response to a variety of environmental stresses as stress-induced PCD activation signifies that damaged cells are unable to cope with the prolonged redox imbalance (Petrov et al., 2015). PCD is activated as the cells’ last act of preservation because of stress-induced oxidative damage to organelles and macromolecules (Wituszynska and Karpinski, 2013). Unlike necrotic death, selective PCD activation improves the overall chances of plant survival as it maintains tissue and organ integrity by eliminating damaged cells that accumulate during stress (Wituszynska and Karpinski, 2013). By eliminating cells in a controlled manner, the remaining plant cells can recycle the metabolic precursors from dying cells to increase the likelihood of cell survival (Hoang et al., 2016).

Stress-induced PCD has broad implications for global agricultural practises as it affects crop yield and productivity (Mittler and Blumwald, 2010). With the advance of rapidly changing climates all over the globe, there is a growing interest in developing methods for attenuating environmental stress-induced PCD to minimise crop yield losses (Kim et al., 2014; Hoang et al., 2016). Consequently, it is important for researchers to have an array of methods available to quantify PCD levels *in vivo*. Current methods rely on either the direct scoring of PCD based on its distinctive cell morphology, or indirectly by tracking PCD-triggering molecular signals (e.g. ROS, intracellular Ca^2+^ levels, and cyclic guanosine monophosphate) (Chen et al., 2018; Doccula et al., 2018; Terrón-Camero et al., 2018) and various mitochondrial markers (Xiao et al., 2018). Other indirect methods for quantifying PCD include the measurement of molecular markers generated under oxidative damage (reactive carbonyl species, DNA and lipid damage) (Mano and Biswas, 2018), or PCD executors such as mitogen-activated protein kinase (MAPK) signalling cascades (Wu and Jackson, 2018) and vacuolar processing enzyme (VPE) activity (Hatsugai and Hara-Nishimura, 2018). All of these methods have a wide range of applications for investigation of the different phases of the plant stress response, but it is important to remember that cells integrate multiple PCD-inducing signals across many different subcellular compartments, and not just a lone signal as measured by the aforementioned methods (Petrov et al., 2015). This was illustrated in work by Kacprzyk et al. (2017) who showed that chemical modulators that alter mitochondrial permeability transition, ATP synthesis and Ca^2+^ signalling also inhibit protoplast retraction in stressed cells, showing that multiple signalling pathways are acting collectively to modulate PCD. Perception of stress cues generates PCD-inducing signals at the endoplasmic reticulum (ER), chloroplast and mitochondria, but each organelle has distinctive mechanisms for processing the signal (Petrov et al., 2015).

The intricate signalling networks modulating PCD emphasises the serious consequences the cellular decision to undergo PCD holds for the survival of the whole organism. Cells regulate PCD by balancing pro- and anti-apoptotic signals, and the decision to live or die depends on which direction the balance shifts. This highlights the biggest difference found between indirect and direct PCD quantification methods. Indirect methods track the progression of molecular markers, signalling networks or metabolic changes that stressed cells undergo, while direct PCD scoring shows the final outcome of the cells decision-making procedure, whether cells stay alive or undergo PCD.

This paper provides evidence that direct *in vivo* PCD scoring is a useful marker of stress tolerance in cereals as it integrates multiple stress inputs (and combinations thereof) to provide a cohesive picture of the stress response. Studies using direct PCD scoring methods generally involve *in vitro* plant cell cultures, but they can be labour intensive to establish and because of divergent mitotic patterns, not all plant species will have the right morphologies to form uniform suspension cultures (Cimini et al., 2018). More importantly, it is pertinent to assess the effects of PCD modulators in the whole plant context, as tissue-specific cells will not respond in a synchronised manner as would be seen in homogenous plant cell cultures (Reape et al., 2015). Given these points, using seedlings as an *in vivo* model system for investigating plant PCD offers a more accurate representation compared to artificially controlled reconstructions using *in vitro* methods (Kacprzyk et al., 2011; Reape et al., 2015). A novel model system involving root hairs for direct PCD scoring was demonstrated by Hogg et al. (2011) as root hairs are lateral single-celled extensions from root epidermal cells, are present in quantities large enough for sample enumeration, and are easily accessible for pharmacological treatment. The protocol developed was termed the root hair assay (RHA) and was used to establish heat stress response curves in *Arabidopsis* seedlings, and Hogg et al. (2011) also successfully extrapolated the assay to *Medicago truncatula*, *Zea mays*, and *Quercus robur* seedlings. Furthermore, Kacprzyk et al. (2014) demonstrated RHA use with genetic and pharmacological tools to assess the signalling networks regulating the PCD response in *Arabidopsis* seedlings.

In this paper, we build on these past works to show that stress-induced PCD levels can be a novel marker for identifying stress tolerance in cereal varieties of *Triticum aestivum* (wheat) and *Hordeum vulgare* (barley). Using the RHA as an early screening tool, we developed a protocol for identifying stress tolerant and susceptible cereal varieties by subjecting <2-day-old seedlings to increasing heat and salt stress intensities. By reviewing the dose-dependent response, we identified the ‘inflection point’ for each species and stress treatment. The inflection points indicate the stress dose which exhibited the largest variances in stress-induced PCD levels and once identified, these inflection points were then used to assess the basal, induced and cross-stress tolerance of wheat varieties by exposing plants to single, combined and multiple individual stresses. Single stress exposure involves the application of a single stress-factor, multiple individual stresses are non-overlapping repetitive stresses at different time-points, while combined stress is two or more stresses applied simultaneously that overlap to a certain degree (Pandey et al., 2017).

Basal tolerance was assessed using single and combined stress exposure as both treatments highlight the intrinsic ability of plants to survive stress by its baseline physiological state without prior stress exposure or acclimation (Arbona et al., 2017). Combined stress treatments are highly distinct from single stress-factor treatments as the former generates a unique stress phenotype that is distinct from the latter (Mittler, 2006; Rasmussen et al., 2013; Rivero et al., 2014). Rasmussen et al. (2013) divided the unique stress phenotype into five categories (prioritized, similar, combinatorial, cancelled and independent), but for simplicity’s sake, we refer to the original stress phenotype categories devised by Mittler (2006) who divided the response into synergistic, antagonistic or neutral interactions, of which all five stress modes fall into (**Supplementary Figure 1A**). Finally, we used multiple individual stresses to study induced and cross-stress tolerance, the phenomenon where the initial stress exposure makes plants more resistant to other stress types (Walter et al., 2013; Rejeb et al., 2014; Pandey et al., 2017). As **Supplementary Figure 1B** illustrates, the first stress cue can either prime (positive and neutral) or predispose (negative) plants to recurrent stress exposure (Pandey et al., 2017). Priming enables plants to reach a new metabolic steady-state higher than its pre-stress levels by reprogramming the metabolome and making epigenetic changes; primed plants either become resistant to the second stress encounter without additive damage (neutral – maintains same steady state), or have improved tolerance (positive – higher metabolic steady state) (Tausz et al., 2004; Walter et al., 2013; Pandey et al., 2017). Conversely if the cell protective mechanisms are insufficient, predisposition makes plants more vulnerable to repetitive stresses because of lagging stress effects (e.g. excessive oxidative damage) that leads to degradation of the metabolic steady state and higher cell death rates (Tausz et al., 2004; Walter et al., 2013). The variety of responses to different stress exposures shown here demonstrates how stress-induced PCD levels can be used to screen for the formation of unique stress phenotypes, while at the same time, allowing examination of how basal, induced and cross-stress tolerance affects cereal survival.

## Materials and Methods

### Seedling Preparation

Three spring barley varieties were provided by Seedtech^®^, while four spring wheat, four winter wheat and four winter barley varieties were supplied by KWS UK^®^. **Table 1** details the list of cereals and their identifier numbers used in these experiments. In temperate climates, spring and winter varieties differ in the season they are sown. Winter varieties require vernalisation in the cold to flower, while spring varieties do not. In barley, the vernalisation response is controlled by two major loci at *VRN-H1* and *VRN-H2*, while spring alleles have deletions in both loci that enables flowering without vernalization (Cockram et al., 2007). A similar scenario occurs in wheat, but five vernalization-responsive genes (*Vrn1–5*) have been identified (Cattivelli et al., 2002), but the three major vernalization genes responsible for vernalization in both wheat and barley are *VRN1*, *VRN2* and *VRN3* (Distelfeld et al., 2009).

**Table 1:** Cereal Variety Identifier, Corresponding Species, Season and Provider.

Seed Identifier	Species	Season	Provider
SB1	*H. vulgare* (Barley)	Spring	Seedtech^®^
SB2			
SB3			
WB1	*H. vulgare* (Barley)	Winter	KWS UK^®^
WB2			
WB3			
WB4			
SW1	*T. aestivum* (Wheat)	Spring	KWS UK^®^
SW2			
SW3			
SW4			
WW1	*T. aestivum* (Wheat)	Winter	KWS UK^®^
WW2			
WW3			
WW4			

SB, spring barley; WB, winter barley; SB, spring wheat; WW, winter wheat.

#### ***T. aestivum*** (Wheat) Seedling Preparation and Germination

Wheat seeds were soaked in sterile distilled water (SDW) at room temperature for 3 h. In a sterile flow cabinet, water was drained from seeds, a 20% bleach solution (Domestos^®^ disinfectant: sodium hypochlorite 4.5 g per 100g) was added, and the mixture was shaken for 4 min and rinsed 5 times with SDW. Using sterile forceps, 10 surface-sterilised seeds were placed between two layers of sterile 10 mm Whatman™ filter paper (pre-soaked with 3 cm^3^ SDW) in a Petri dish. Seeds were arranged far apart from one another to prevent roots from tangling after germination to minimise root hair damage. Plates were sealed with Parafilm, wrapped in foil and stratified at 4°C for at least two days to synchronise germination. To germinate seeds, plates were placed in a 21°C growth chamber (light regime: 33 µmol m^-2^ s^-1^, 16-h light: 8-h darkness) and used for stress assays after 1 day of growth.

#### ***H. vulgare*** (Barley) Seedling Preparation and Germination

The procedure to prepare barley seedlings for testing was similar to the protocol used for wheat seedlings; however, barley seeds were left to grow for 2 days as initial testing (data not shown) showed inadequate germination levels after 1 day of growth.

### Stress Application and Scoring of Cell Modes

Barley and wheat seedlings were transferred into Petri dishes under aseptic conditions with care to prevent mechanical damage which would inflate the background death levels of root hairs. SDW (2 cm^3^) was pipetted into the germination plates and swirled to dislodge the roots from the filter paper. Seedlings were transferred to Petri dishes (containing 25 cm^3^ SDW), heated for 10 min in a water bath at specific temperatures (25, 35, 45, 50, or 55°C) and returned to the 21°C growth chamber. Viability and cell death (PCD and necrosis) were scored 14–16 h after stress application to allow PCD morphology to develop fully as per Hogg et al. (2011).

The longest root in 1-day-old wheat seedlings was counted as shorter roots lacked sufficient root hair density for accurate cell mode enumeration. In contrast, 2-day-old barley seedlings have multiple roots (3–5) of approximately equal length. Preliminary RHA testing (data not shown) showed that barley roots on the same seedling have similar viability, PCD, and necrosis levels therefore because of the insignificant variability of roots from the same plant sample, subsequent heat stress curves only involved the enumeration of one root per barley seedling.

To score cell mode, the seedlings were stained with a 0.001% w/v fluorescein diacetate (FDA) solution for 2 min and examined using an Olympus BX61 microscope under a mercury lamp with a fluorescein isothiocyanate (FITC, wavelength 485 nm) filter. Using a combination of viability staining and cell death morphology, root hairs were scored as viable if they were fluorescent (FDA positive), PCD if they had a retracted cytoplasm and negative FDA stain, and necrotic if they did not possess a retracted cytoplasm and negative FDA stain. **Supplementary Figure 2A** depicts the different cell mode morphologies found in FDA-stained stressed and unstressed root hairs of *Arabidopsis thaliana*, the model organism in which the RHA was originally developed. Cereal root hairs display similar morphologies when viable, PCD or necrotic, but are longer and occur more frequently along the main root compared to *A. thaliana*. Consequently, it is difficult to take clear images of cereal roots depicting the different cell morphologies; hence we use *A. thaliana* images here to clearly illustrate cell death morphology in individual root hair cells. **Supplementary Figure 2B** shows cell death morphology in wheat root hairs but at a lower magnification. These are included because on occasion, salt-stressed wheat seedlings displayed mixed markers (retracted cytoplasm and FDA positive) because of plasmolysis (**Supplementary Figure 2B**). Under these circumstances, root hairs displaying mixed markers were rinsed with SDW and remounted on microscope slides without additional FDA staining. This removes excessive background FDA staining and makes it easier to distinguish between viable (strong fluorescence) and PCD (weak, almost imperceptible fluorescence) root hairs. At least 100 root hairs were scored per seedling across both sides of the primary root to provide an accurate representation of viable, PCD and necrosis levels. Each cell mode result is depicted as the percentage of cell mode over total number of root hairs, where viability% + PCD% + necrosis% = 100%.

#### Establishing Salt Stress Response Curves in ***T. aestivum*** Seedlings

1-day-old wheat seedlings were placed in Petri dishes filled with 25 cm^3^ NaCl (50, 100, 150, 200 or 250 mM) for 5 min, before being transferred into new Petri dishes containing 25 cm^3^ SDW. Seedlings were returned to the 21°C growth chamber and scored 14-16 h after stress application.

#### Evaluating the Single, Combined and Multiple Stress Responses of ***T. aestivum*** Seedlings to Heat and Salt Stress

**Figure 1:**
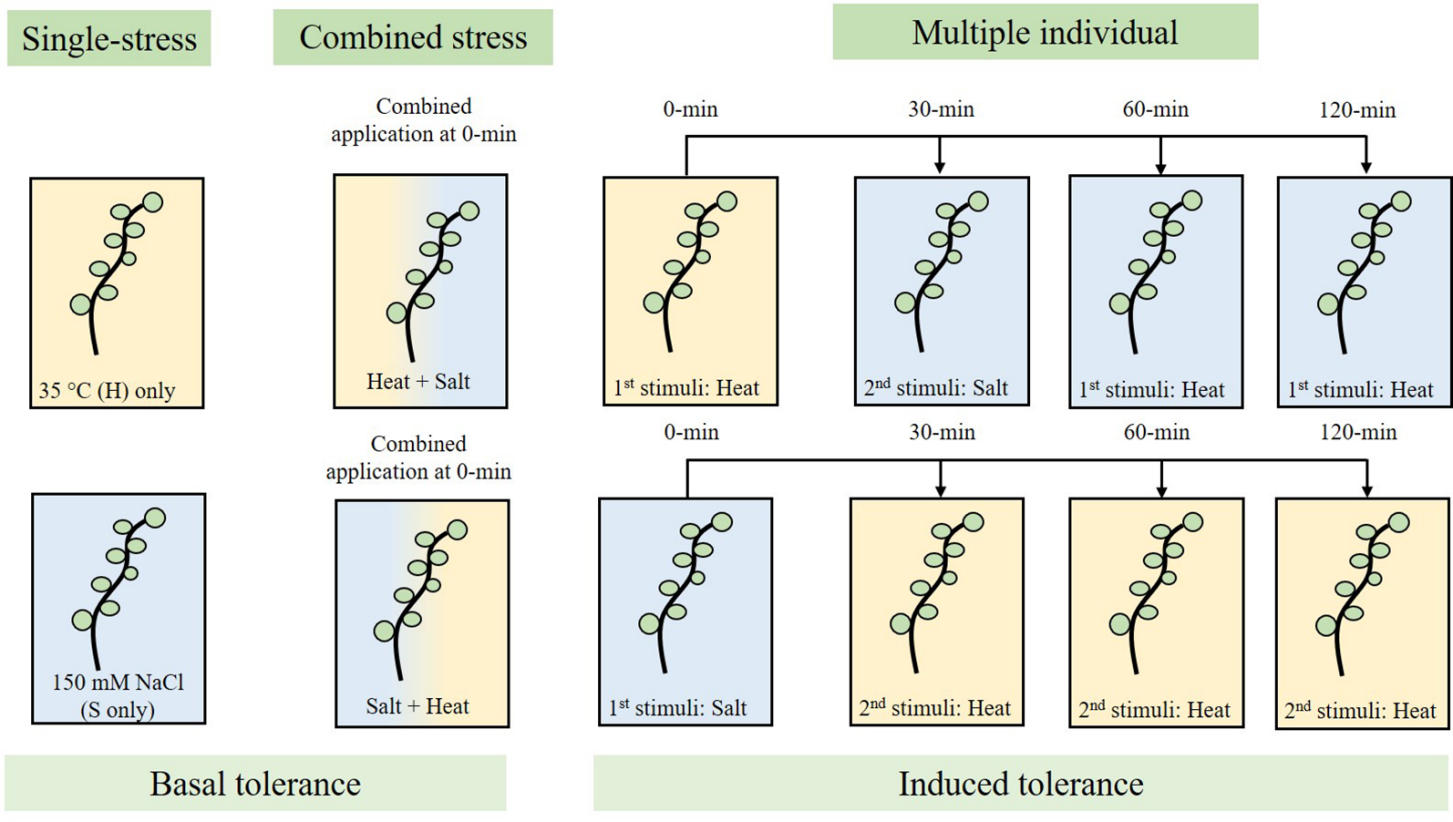
Experimental workflow used to assess basal stress tolerance (single and combined), and induced stress tolerance (multiple individual) in wheat seedlings in response to 35°C heat (H) and/or 150 mM NaCl salt (S) stress. Single-stress involves the application of a single stress-factor (H only, or S only), combined stress involved the overlapping application of 35°C heat followed by salt stress (H+S; 0-min) and vice versa (S+H; 0-min), and multiple individual testing involves the application of the first stress stimuli (0-min), followed by application of the second stress stimuli at 30, 60, and 120-min.

Eight wheat varieties were examined for their response to single, combined and multiple stresses. As a result of identifying the 35°C heat and 150 mM NaCl inflection points, 1-day-old wheat seedlings were subjected to heat (35°C) and/or salt (150 mM NaCl) stress at specific time-intervals. In the first data-set, samples were subjected to 35°C stress for 10 min at the 0-min mark, followed by 150 mM NaCl stress for 5 min at the 30, 60 and 120-min mark, followed by transfer into 25 cm^3^ SDW. In the second data-set, samples were subjected to 150 mM NaCl stress for 5 min at the 0-min mark, transferred into SDW-containing plates, and followed by 35°C heat stress for 10 min at the 30, 60 and 120-min mark. **Figure 1** summarises the process used to examine basal and induced tolerance using single, combined and multiple individual stresses. Controls include single-stress (35°C only, or 150 mM NaCl only) and double-stress (heat and then salt (H+S) or, salt and then heat (S+H) at the 0-min mark).

### Statistical Analysis

IBM^®^ SPSS^®^ Version 24 (RRID : SCR_002865) was used to analyse results for significant changes (*p* < 0.05) across stress treatments and cereal (barley and wheat) varieties. Statistical tests used include one-way ANOVA (Tukey or Dunnett Post-hoc Test), bivariate analysis (Pearson’s correlation), and independent-samples *t*-test.

## Results

### Thermotolerance of *H. vulgare* Varieties

**Figure 2:**
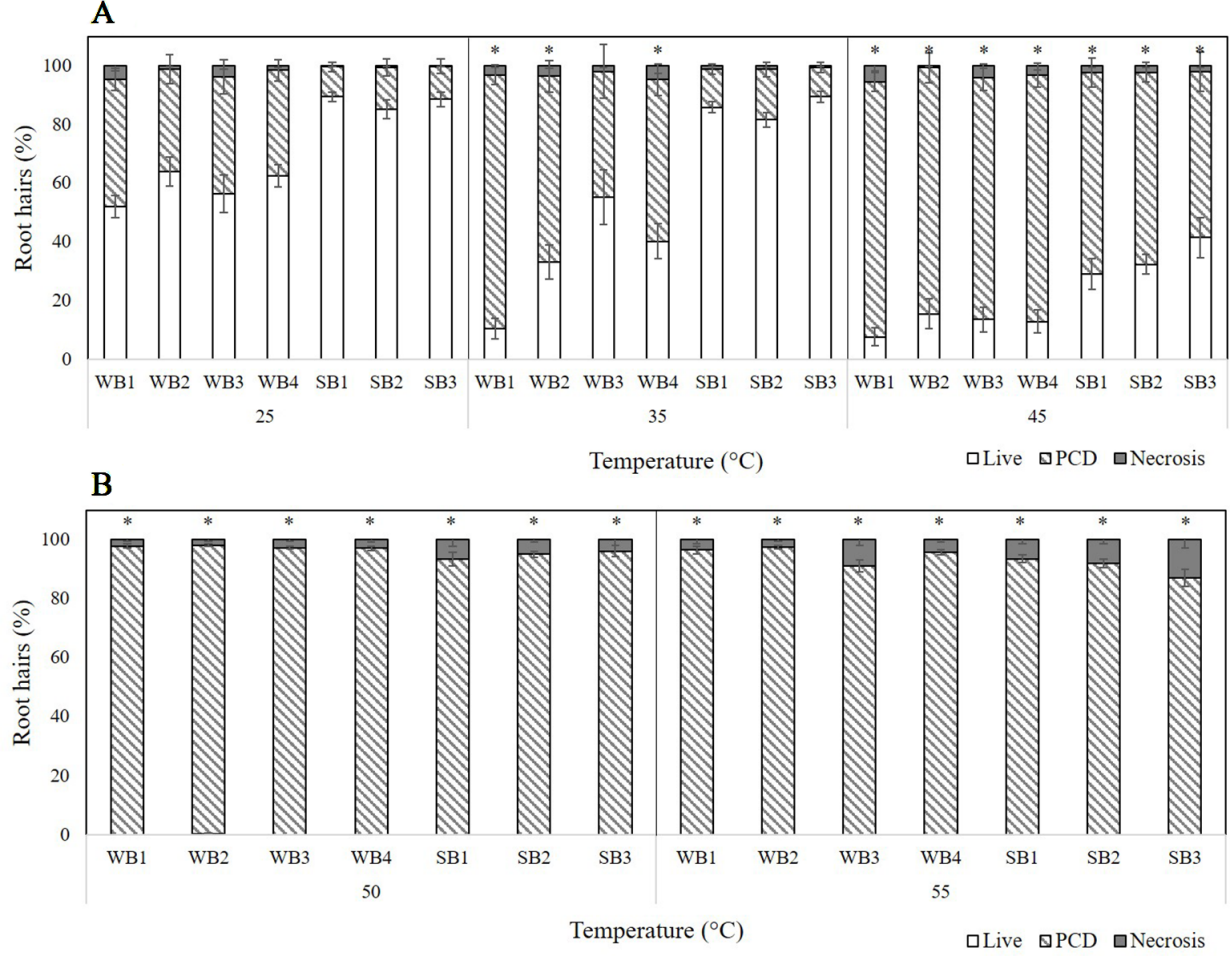
Effect of **(A)** low-to-medium or **(B)** high heat stress on root hair viability and cell death (PCD and necrosis) levels in varieties of winter (WB1-4) and spring (SB1-3) barley. (*) marks PCD results significantly (*p* < 0.05) different from the 25°C dataset, using a one-way ANOVA Dunnett post-hoc test (**Supplementary Table 1**). Error bars = standard error of n ≥ 8 replicates.

Four winter barley (WB) and three spring barley (SB) varieties were tested for their thermotolerance by stressing 1-day-old seedlings for 10 min at temperatures ranging from 25 to 55°C. Based on the changing cell mode ratios across the temperature gradient, three threshold stress-responses were observed: 1) stress-tolerant (25°C) where PCD levels were at their lowest (and necrosis levels were negligible), 2) the viable/PCD ‘inflection point’ (35°C), and 3) the PCD zone (45–55°C) where the majority of root hairs died by PCD. We observed a clear distinction between spring and winter varieties as all three spring varieties had consistently lower PCD levels at low heat stress (25–35°C) compared to their four winter counterparts. The PCD levels of the spring barley varieties remained stable (10–17%) across 25°C and 35°C heat stress, unlike the winter varieties which increased when heat stress was increased from 25°C (35–40%) to 35°C (43–63%). Statistical analysis confirmed these observations: PCD levels only changed significantly (*p* < 0.05) in WB1, WB2, and WB4 seedlings when heat stress was increased from 25°C to 35°C but remained stable in the remaining varieties (**Supplementary Table 1**). A similar trend was noted at medium heat stress (45°C) where spring varieties remained more resistant to heat shock, with average PCD levels of 63%. In contrast, PCD levels of all four winter barley varieties were significantly higher (83–87%) at 45°C. At high stress (50–55°C), no difference was observed between winter and spring varieties as viability levels declined to ∼0%, with PCD being the predominant cell death mode across all varieties. **Figure 2A** illustrates the clear thermotolerance differences between seasonal varieties; at low-to-medium heat shock, spring varieties were heat-tolerant, but winter barley varieties were heat-susceptible. Stress-induced PCD was the predominant cell mode across all varieties at 45°C, while necrosis levels were generally unchanged at temperatures up to 50°C, but started to increase at 55°C in WB3 and SB3 (**Figure 2B**).

### Stress Tolerance of *T. aestivum* Varieties

#### Thermotolerance of *T. aestivum* Varieties

**Figure 3:**
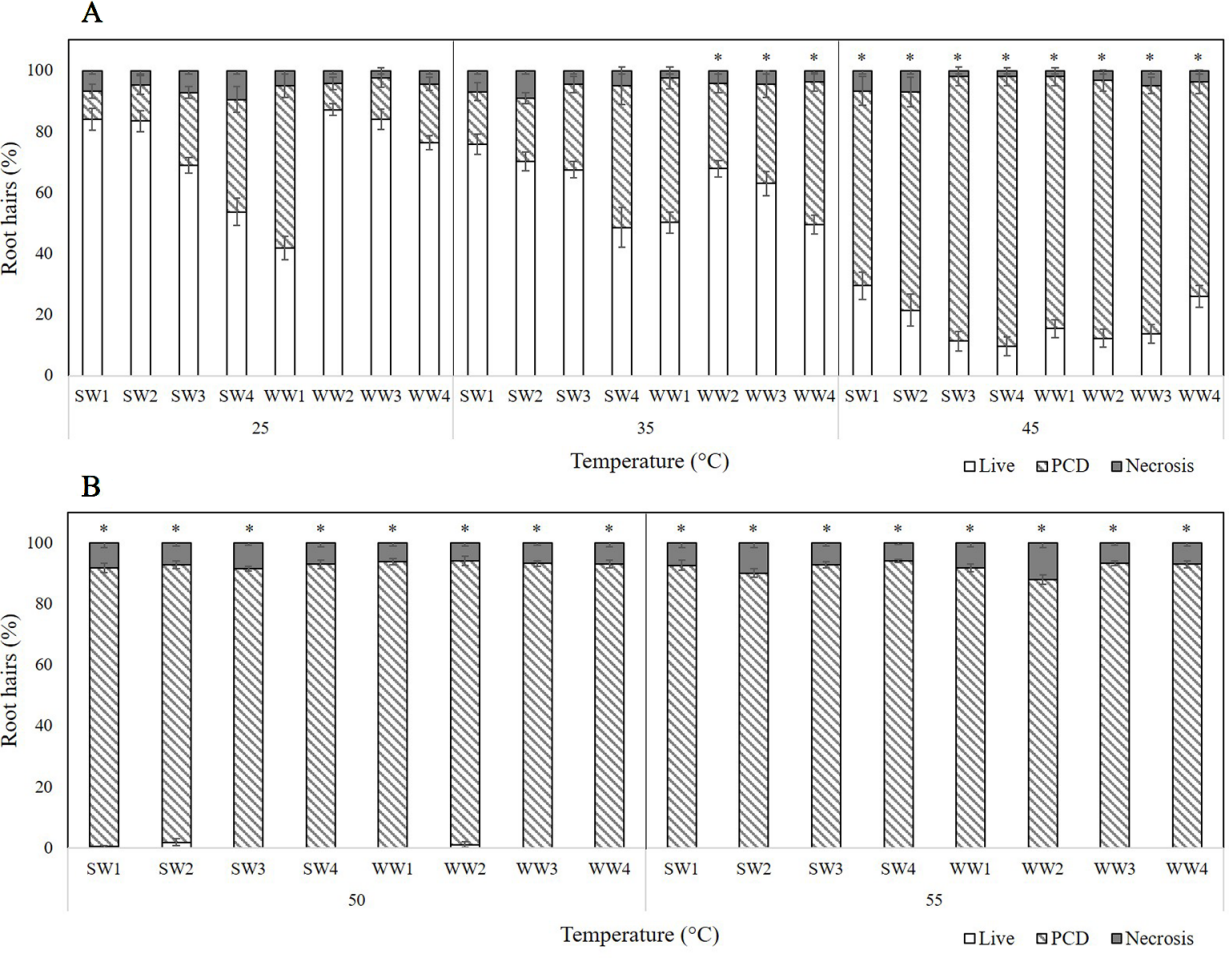
Effect of **(A)** low-to-medium or **(B)** high heat stress on root hair viability and cell death (PCD and necrosis) levels of four spring wheat (SW1-4) and four winter wheat varieties (WW1-4). (*) marks PCD results significantly (*p* < 0.05) different from the 25°C dataset, using a one-way ANOVA Dunnett post-hoc test (**Supplementary Table 2**). Error bars = standard error of n ≥ 12 replicates.

Four spring wheat (SW) and four winter wheat (WW) varieties were tested for their resilience to transient heat stress (**Figure 3**) and, again, three stress-response thresholds were identified: stress tolerant (25°C), viable/PCD inflection point (35°C), and the PCD zone (45–55°C). However, unlike the barley varieties, mixed tolerance was seen across both spring and winter varieties of wheat. At low heat stress (25°C), WW1 had the highest PCD levels (53.2%), followed by SW4 (36.8%) and SW3 (23.9%). We observed a comparable trend at 35°C as SW4, WW1, and WW4 had the highest PCD (46–47%) of all the varieties, with limited variance in viability and necrotic levels.

Distinctions between the thermotolerance of wheat varieties were detected as early as 35°C, which was determined as the viable/PCD inflection point; apart from WW2, WW3 and WW4 whose PCD levels rose significantly (p < 0.05) as heat shock increased from 25°C to 35°C, the remaining varieties maintained similar PCD levels (**Supplementary Table 2**). At higher heat stress (45°C), variations in PCD receded as most wheat varieties had ∼80% PCD, although SW1, SW2 and WW4 lines still exhibitied remarkble heat resistance, with stress-induced PCD ranging from 63 to 71%. Beyond this point, viability declined to ∼0%, with PCD remaining the primary death mode at 50°C and 55°C. Even at 55°C heat shock, necrotic levels remained remarkably stable across the wheat varieties and temperature gradient, apart from WW1 and WW2 seedlings that had a 2 to 3-fold increase in necrosis, compared to that seen at the 50°C data-point.

#### Evaluating *T. aestivum* Varieties for Salt Tolerance

**Figure 4:**
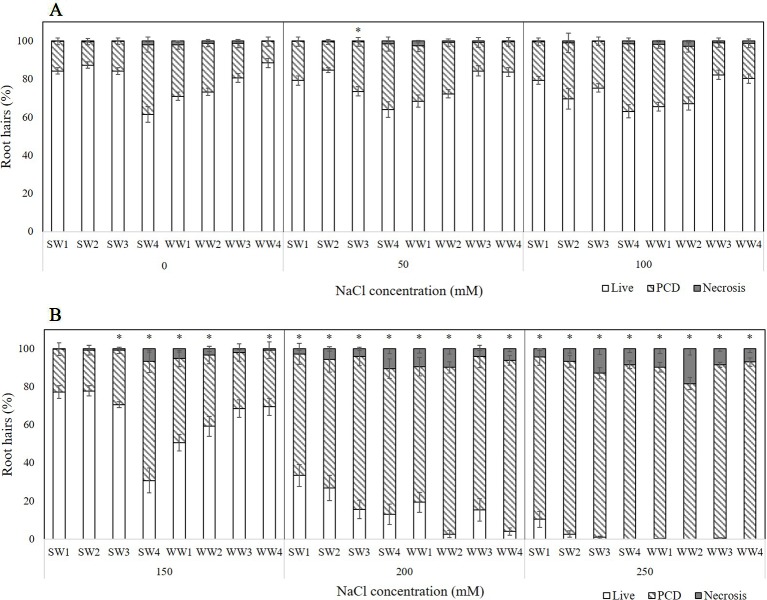
Effect of **(A)** low or **(B)** medium-to-high salt stress on root hair viability and cell death (PCD and necrosis) levels of four spring wheat varieties (SW1-4) and four winter wheat varieties (WW1-4). (*) marks PCD results significantly (*p* < 0.05) different from the 0 mM NaCl (i.e. SDW control) dataset, using a one-way ANOVA Dunnett post-hoc test (**Supplementary Table 3**). Error bars = standard error of n ≥ 12 replicates.

Four spring wheat (SW1-4) and four winter wheat (WW1-4) varieties were tested for their tolerance to transient salt stress (**Figure 4**). Three stress-response thresholds were also detected: stress-tolerant (50–100 mM NaCl), the viable/PCD inflection point (150 mM NaCl), and the PCD zone (200–250 mM NaCl). At low salt stress (50–100 mM), we could clearly see the distinctions between the salt-tolerant and salt-susceptible varieties: SW1, SW2, WW3, WW4 were identified as the salt-tolerant lines as they had the lowest stress-induced PCD (15–20%) of all the varieties. Discrepancies became even larger when effects were examined at 150 mM NaCl (viability/PCD inflection point); PCD predictably increased across all varieties but SW4, WW1, and WW2 had elevated PCD levels compared to the other varieties tested. WW1 and WW2 had PCD ranging from 37-44%, while SW4 had almost double PCD (62.6%) which equates to a 27.2% increase from its nearest 100 mM data-point (**Figure 4A**). The remaining five varieties had similar PCD ranging from 21–30%. Beyond this point, medium salt stress (200–250 mM) caused PCD to become the predominant cell mode over viable and necrotic cells. Interestingly, SW1 and SW2 still had the lowest PCD levels at 200 mM NaCl (64–68%), indicative of their salt tolerance, since the average PCD across the other varieties was 80.9%. Nevertheless, this discrepancy disappeared at higher 250 mM NaCl doses as PCD (85-93%) became similar across all eight varieties (**Figure 4B**). Necrosis levels did not change significantly in the experiment.

#### Screening *T. aestivum* Varieties for Dual Stress Tolerance

The discovery of the three distinct stress-response phases across all the heat and salt stress gradients tested in wheat prompted the preparation of a tolerance matrix (**Table 2**) to determine if varieties displayed dual tolerance to both heat and salt stress. As previously stated, the largest deviations in stress-induced PCD levels arise at the inflection point, making it easier to compare differences in the tolerance strength of the varieties. While we do see fluctuations at the other phases, stress-induced PCD levels tend to cluster too closely to pick out subtle variations between the investigated varieties. For example, PCD is generally low in the stress-tolerant zone, but predominantly high in the PCD zone. Consequently, we focused on performance at the viable/PCD inflection point to identify stress-tolerant or susceptible varieties.

**Table 2:** Tolerance matrix examining the tolerance or susceptibility of wheat seedlings to salt or heat stress at different stress-response phases (stress-tolerant, viable/PCD inflection point and PCD zone) highlights SW1 and SW2 as stress tolerant varieties. Bivariate analysis (Pearson) found correlation between PCD levels of heat and salt-stressed seedlings in the stress-tolerant phase (n = 105) and viable/PCD inflection point (n = 115), but not in the PCD zone (n = 121).

Stress-response Phase	Stress Applied	Variety							
		SW1	SW2	SW3	SW4	WW1	WW2	WW3	WW4
**Stress-tolerant**	**25 °C**	++	++	x	x	x	++	+	+
	**PCD (%)**	9.1	11.86	23.86	36.84	53.21	8.66	13.68	19.19
	**50 mM NaCl**	+	++	x	x	x	x	++	++
	**PCD (%)**	20.4	15	26	34.4	29	26.9	15	15.9
	Pearson's correlation coefficient = 0.223*, *p*-value = 0.022, R^2^ linearity = 0.050								
**Viable/PCD inflection point**	**35 °C**	++	++	+	x	x	+	+	x
	**PCD (%)**	17.3	20.7	28	46.5	47.4	28	32.7	46.7
	**150 mM NaCl**	++	++	+	x	x	x	+	+
	**PCD (%)**	22.5	21.5	28.5	62.6	44.3	37.3	29.4	29.7
	Pearson's correlation coefficient = 0.333*, *p*-value = 0.000, R^2^ linearity = 0.111								
**PCD zone**	**45 °C**	++	+	x	x	x	x	x	+
	**PCD (%)**	63.8	71.5	86.9	88.3	82.6	84.5	81.3	70.3
	**200 mM NaCl**	++	++	x	+	+	x	x	x
	**PCD (%)**	63.8	67.6	80.1	76.5	71.1	87.4	80.5	89.7
	Pearson's correlation coefficient = -0.015, *p*-value = 0.867, R^2^ linearity = 2.365 x 10^-4^								

Key: ‘X’ = Stress-susceptible, ‘++’ = stress-tolerant, ‘+’ = moderately stress-tolerant.

**Figure 5:**
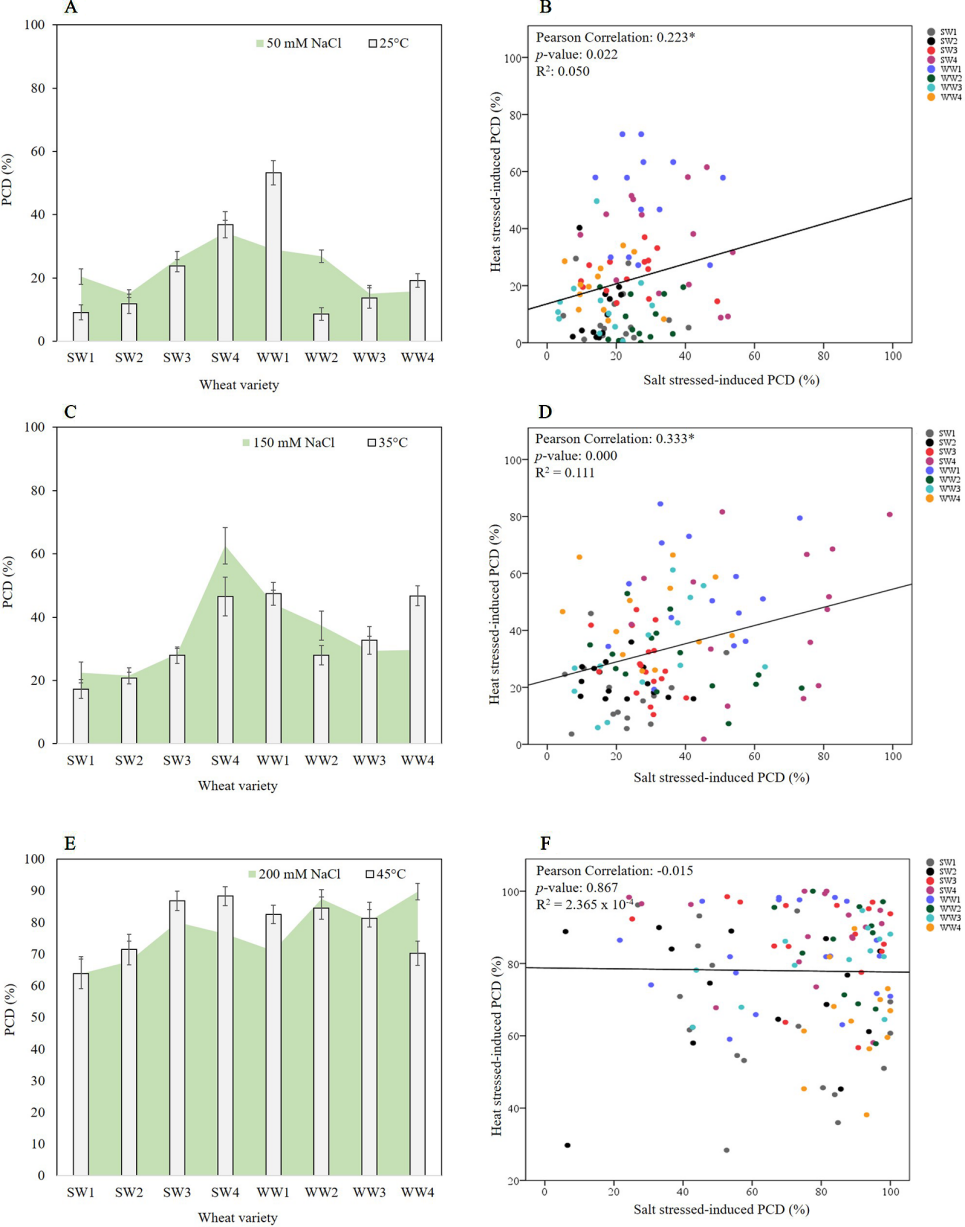
Pearson correlation analysis between the tolerance/susceptibility of wheat varieties to salt and heat stress. Figures on the left column represent overlaid heat and salt datasets, while their respective scatterplots are shown on the right column. **(A**, **B)** Stress-tolerant phase of 25°C and 50 mM NaCl, **(C**, **D)** the viable/PCD inflection point of 35°C and 150 mM NaCl, and **(E, F)** the PCD zone of 45°C and 200 mM NaCl. Correlation was found between PCD levels of heat and salt-stressed seedlings in the stress-tolerant phase (0.223, *p*-value = 0.022 and n = 105) and viable/PCD inflection point (0.333, *p*-value = 0.000 and n = 115), but not in the PCD zone (-0.015, *p*-value = 0.867 and n = 121).

The first stress-tolerant threshold (25°C; 50 mM NaCl) denotes the phase where the cell protective mechanisms are enough to repair oxidative damage therefore cells maintain high viability and low PCD levels. Bivariate analysis was used to measure the strength of association between heat and salt stress-induced PCD levels. We found statistically significant (*p* < 0.05) correlation between both variables, with a Pearson’s correlation coefficient of 0.223 (n = 105), showing that PCD levels in heat shocked seedlings correlated with their salt-stressed counterparts. As illustrated in **Figures 5A, B** and **Table 2**, low salt tolerance was observed in SW3, SW4, WW1, and WW2 seedlings, with PCD ranging from 26–34%, compared to the remaining seedlings exhibiting PCD of 15-20%. Similar varieties were also found to be susceptible to minimal (25°C) heat stress, as elevated PCD levels were found in SW3 (23.9%), SW4 (36.8%) and WW1 (53.2%), and to a certain extent, WW4 (19.2%). PCD in the four remaining varieties was substantially different and averaged 10.8%.

A similar trend was observed at the viable/PCD inflection point (35°C; 150 mM NaCl) shown in **Figures 5C, D** and **Table 2**. Compared to the stress-tolerant thresholds, a stronger correlation was noted here as we detected a highly statistically significant correlation of 0.333 (*p* < 0.01) between PCD levels of heat and salt-shocked seedlings (n = 115). At 150 mM NaCl, the highest PCD values were seen in SW4 (62.6%), WW1 (44.3%) and WW2 (37.3%), while the PCD levels in the other lines only ranged between 21-30%; the lowest PCD levels at 150 mM NaCl were seen in SW1 and SW2 which had ∼22% PCD. Under 35°C heat stress, elevated PCD (∼47%) was seen in SW4, WW1 and WW4, whereas the lowest PCD levels were seen in SW1 (17.3%) and SW2 (20.7%). Collectively, these results show that similar wheat varieties displayed dual tolerance (SW1 and SW2) or susceptibility (SW4 and WW1) to independent heat and salt stress.

Finally, no significant correlation (-0.015, where *p* > 0.05 and n = 121) was found at the PCD zone (45°C; 200 mM NaCl) between heat and salt-stressed seedlings (**Figures 5E, F** and **Table 2**). At this stage, both stress intensities were high enough to overcome most of the differences in basal tolerance between wheat varieties; apart from SW1 and SW2 that maintained PCD levels lower than 70% at 200 mM NaCl (**Figures 5E, F**), the remaining six varieties averaged 80.9%. Similarly, at 45°C, SW1, SW2, and WW4 had the lowest PCD (64-72%), while the other five lines had PCD levels >81%.

### Evaluation of *T. aestivum* Varieties for Basal, Induced and Cross-Stress Tolerance to Heat and Salt Stress

**Figure 6:**
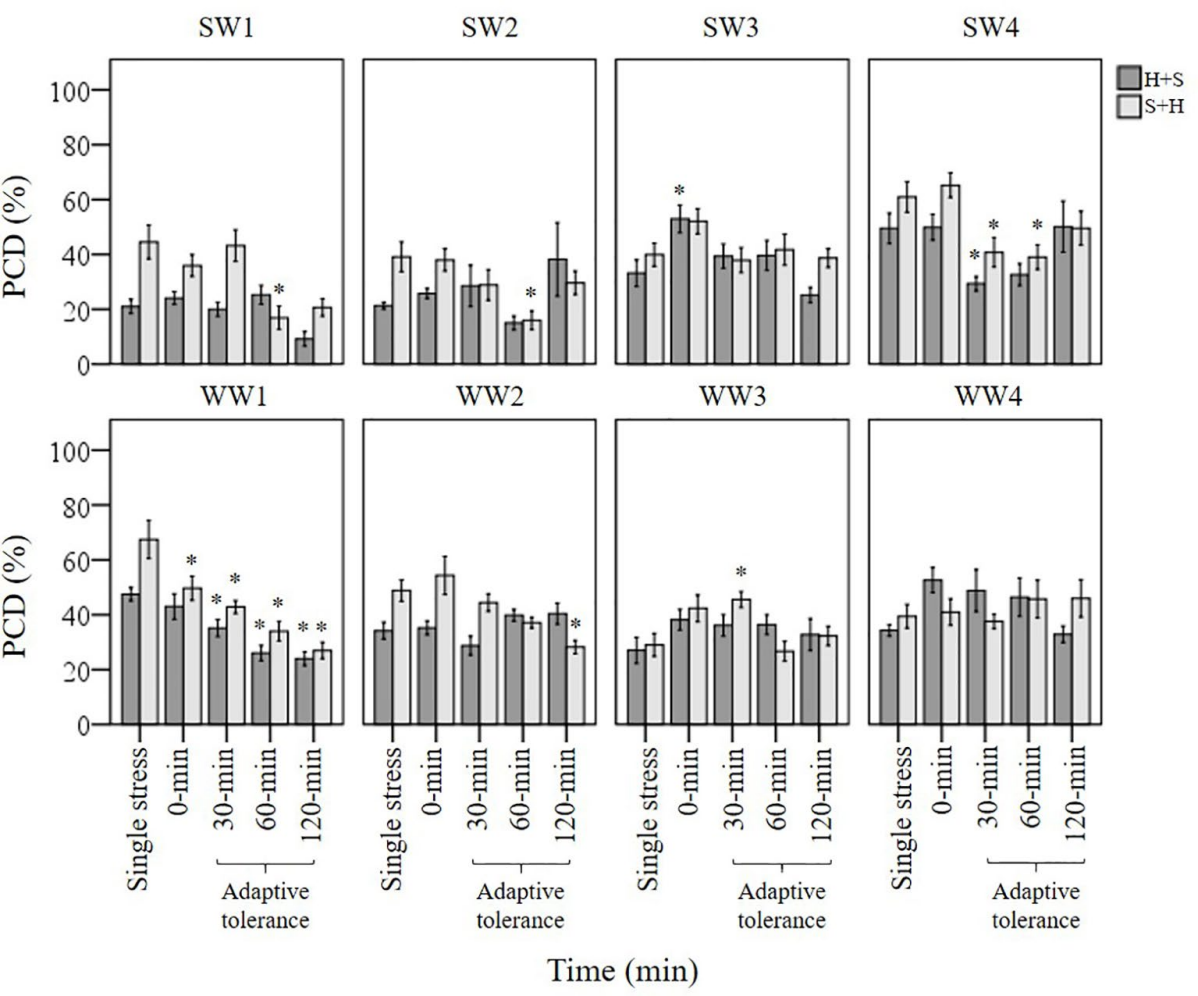
Examining how single, combined and multiple individual stress exposures affects stress-induced PCD in wheat varieties. The initial stress cue (35°C heat or 150 mM NaCl) is applied at the 0-min mark, followed by the second stress application at different time-points (30, 60 and 120-min). (H+S) refers to heat stress as the initial cue, followed by salt stress, while (S+H) refers to salt stress as the first cue, followed by heat stress at the relevant time-points. (*) marks PCD results significantly (*p* < 0.05) different from the single-stress factor control (H-only or S-only), using a one-way ANOVA Dunnett post-hoc test (**Supplementary Table 4**). Error bars = standard error of n ≥ 4 replicates.

**Figure 7:**
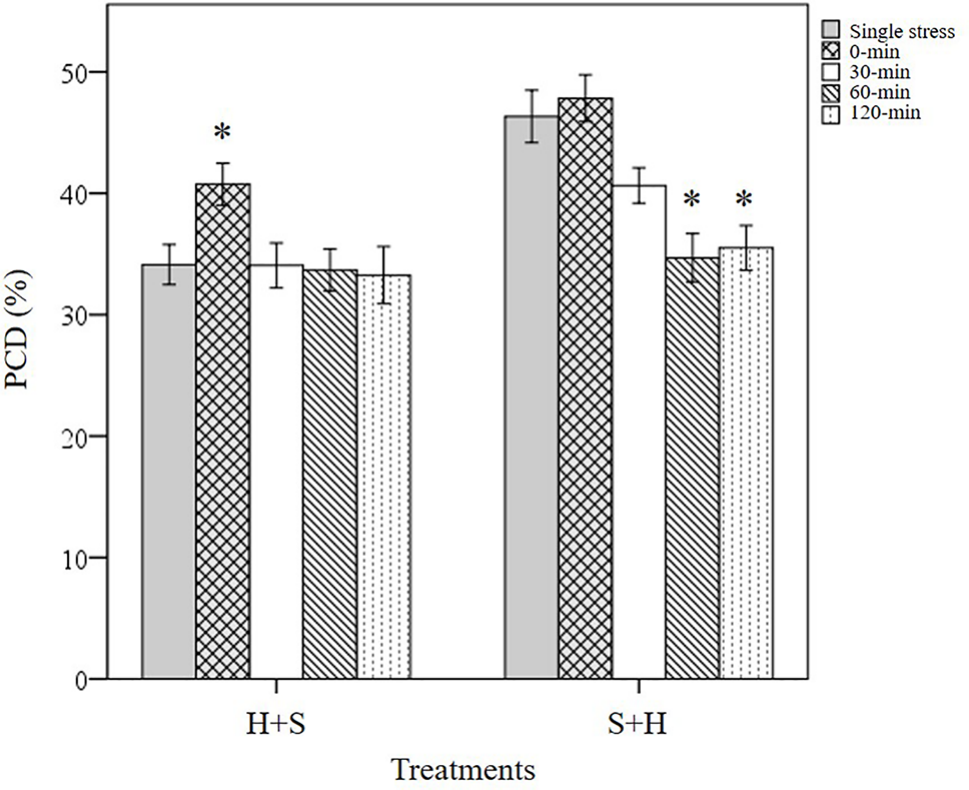
Overall trends noted in stressed wheat seedlings by varying the initial stress cue. (*) marks PCD results significantly (*p* < 0.05) different from the single-stress factor control (H-only or S-only), using a one-way ANOVA Dunnett post-hoc test (**Supplementary Table 5**). Values represent the average PCD levels across the eight varieties, where error bars = standard error of n ≥ 89 replicates.

Three types of stress exposure were investigated in this final study: single, combined and multiple individual stresses. Basal tolerance of the seedlings was examined at the viable/PCD inflection point by applying a single (35°C heat or 150 mM NaCl) or combined stress (simultaneous application of heat and salt at the 0-min time-point). The adaptive tolerance was evaluated by administering the first stress trigger (heat or salt) at the 0-min mark, followed by the second stress across three time-points (30, 60 and 120 min). **Figure 6** depicts how each individual wheat variety responds to unique stress exposures as a function of their stress-induced PCD levels. Given that basal tolerance reflects the genetically pre-determined ability to withstand stress without prior exposure (Arbona et al., 2017), SW1 and SW2 were identified as varieties with high basal tolerance, while SW4 and WW2 were singled out as varieties with low basal tolerance, based on their performance against single and combined stress treatments (see section: *T. aestivum Cross-Stress Tolerance Depends on the Initial Stress Cue*). Interestingly, varieties with high basal tolerance (SW1 and SW2) had a slow induced tolerance response, unlike stress-susceptible SW4 and WW1 which adapted faster, as elaborated in the section *Individual T. aestivum Varieties Under Combined Stress Exposure Exhibit Varying Stress Responses*. By varying the initial stress cue, we observed a few interesting overall trends not immediately apparent from the data presented in **Figure 6**. For that reason, we merged the average stress-induced PCD levels of all eight varieties across the H+S, and S+H datasets (**Figure 7**) and the following trends were revealed. First, cross-stress tolerance experiments showed that stress acclimation and priming were the predominant responses when seedlings were either first heat or salt-shocked, respectively. Second, under combined stress, seedlings that were first salt-shocked had similar PCD levels (47.8%) as the single stress-factor control (46.3%), but initially heat-shocked seedlings had statistically higher (*p* < 0.05) stress-induced PCD (40.8%) compared to the heat stressed only dataset (34.1%). Finally, salt stress had a dominating effect over heat stress, and that initial salt shock had a lagging PCD-suppressing effect.

#### *T. aestivum* Cross-Stress Tolerance Depends on the Initial Stress Cue

**Figure 8:**
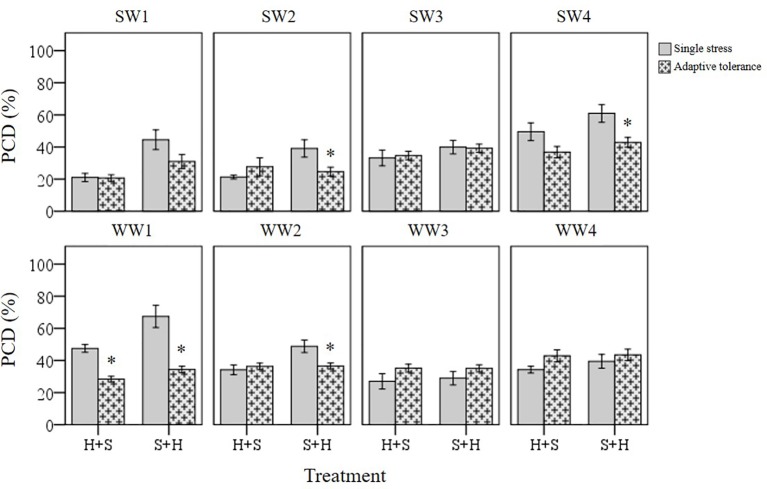
Induced tolerance changes across individual wheat varieties and different initial stress cues. (*) marks PCD results significantly (*p* < 0.05) different from the single-stress factor control (H-only or S-only), using independent *t*-tests (**Supplementary Table 6**). Induced tolerance values represent the merged PCD levels across 30, 60 and 120-min datasets. Error bars = standard error of n ≥ 4 replicates.

Cross-stress tolerance was evaluated in terms of priming (lower PCD levels), acclimation (neutral PCD levels) and predisposition (higher PCD levels) to the second applied stress type, compared to their respective single stress-factor datasets (**Figure 8**). When heat was applied at the first stimuli and followed by subsequent NaCl shock, only WW1 (*p* < 0.05) were grouped under the primed category, while the remaining varieties fell under the acclimation category. However, wheat varieties responded differently when they were first subjected to NaCl shock, followed by later heat stress. Despite maintaining identical stress doses, the varieties were re-shuffled into different categories: primed (SW2, SW4, WW1, and WW2) and acclimation (SW1, SW3, WW3, and WW4). Primed seedlings had statistically lower (*p* < 0.05) PCD levels compared to their respective S-only controls. Predisposition was not observed across both datasets, regardless of the initial stress cue. Thus, stress acclimation was the primary response (87.5%) when heat-shocked wheat varieties were assessed for their cross-stress tolerance to subsequent salt stress. Conversely, priming shared equal dominance (50%) with the acclimation mode when varieties were initially salt-shocked, even though identical stress doses were maintained.

The tendency for specific stress responses based on the initial stress cue (e.g. stress acclimation in H+S; priming in S+H) is illustrated in **Figure 7**, which depicts the average PCD values of all the varieties across both H+S and S+H datasets. When heat shock was the initial stress-cue, similar PCD levels (33-34%) were noted between the single heat stress-factor and the multiple stress (30, 60 and 120-min) dataset. This shows that additional salt stress did not negatively affect previously heat-shocked seedlings (*p* > 0.05), i.e. seedlings were stress-acclimatised against recurrent exposure. However, a different stress response pattern emerged when salt stress was the initial stress cue; exposure to NaCl successfully primed seedlings against subsequent heat damage as we recorded statistically lower (*p* < 0.05) PCD levels at the 60 and 120-min datasets compared to the single NaCl stress-factor dataset. Our results highlights the intricacy of supplying stresses in unique combinations as initial exposure to different stress cues causes divergent responses (**Table 3A**), despite exposure to identical stress dosages.

**Table 3:** Stress matrix summarizing the effect of (A) cross-stress tolerance and (B) the combined stress in response to heat and salt shock in wheat varieties. Symbols (+) denote a reduction in stress-induced PCD levels, (= ) no substantial PCD changes and (-) a net rise in PCD levels from their respective single stress-factor controls.

A) Cross-stress tolerance									
Treatment		SW1	SW2	SW3	SW4	WW1	WW2	WW3	WW4
H+S	Phenotype	=	=	=	=	+	=	=	=
	%PCD difference from H-only control	0%	7%	2%	-13%	-19%	2%	8%	9%
	p-value	0.926	0.278	0.785	0.064	0.001*	0.570	0.155	0.051
S+H	Phenotype	=	+	=	+	+	+	=	=
	%PCD difference from S-only control	-14%	-14%	-1%	-18%	-33%	-12%	6%	4%
	p-value	0.086	0.038*	0.894	0.010*	0.001*	0.012*	0.216	0.476
+ *priming, ‘ = ‘ stress acclimation and ‘-’ predisposition*.									
(B) Combined stress interactions									
Treatment		SW1	SW2	SW3	SW4	WW1	WW2	WW3	WW4
H+S	Phenotype	*=*	*=*	*-*	*=*	*=*	*=*	*=*	*-*
	%PCD difference from H-only control	3%	5%	20%	0%	-4%	1%	11%	19%
	*p*-value	0.389	0.066	0.009*	0.959	0.401	0.8	0.085	0.002*
S+H	Phenotype	*=*	*=*	*=*	*=*	*+*	*=*	*-*	*=*
	%PCD difference from S-only control	-9%	-1%	12%	4%	-17%	5%	13%	2%
	*p*-value	0.253	0.873	0.064	0.553	0.044*	0.496	0.049*	0.818

‘+’ synergistic, ‘ = ‘ neutral and ‘-’ antagonistic interactions.

#### Individual *T. aestivum* Varieties Under Combined Stress Exposure Exhibit Varying Stress Responses

**Figure 9:**
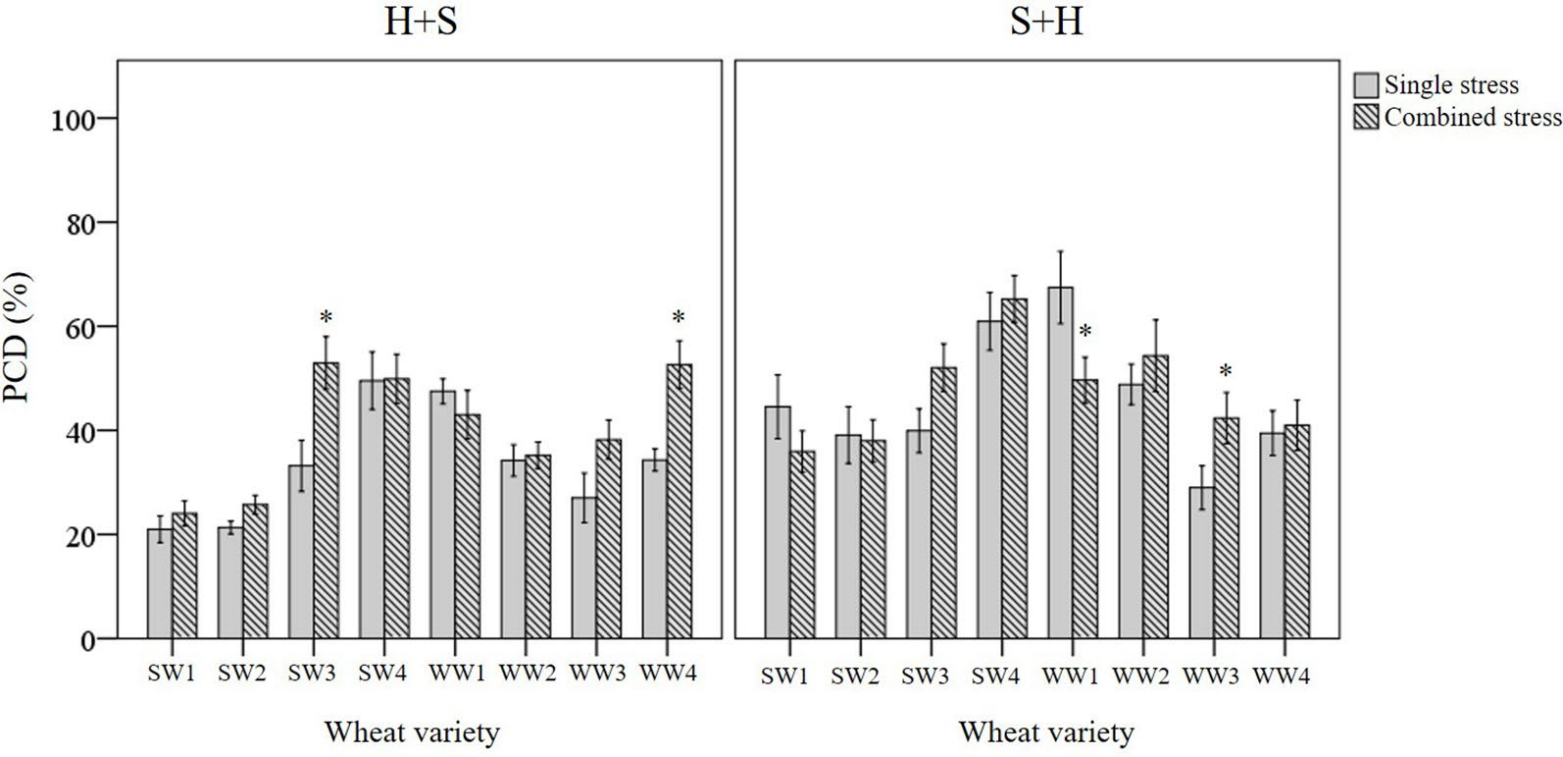
Examining how basal tolerance varies across wheat varieties and different initial stress cues. (*) marks PCD results significantly (*p* < 0.05) different from the single-stress factor control (H-only or S-only), using independent *t*-tests (**Supplementary Table 7**). Combined stress PCD levels reflect the data recorded after simultaneous stress exposure (H+S or S+H) at the 0-min mark. Error bars = standard error of n ≥ 4 replicates.

Basal tolerance to combined stress was assessed by examining the interactions between heat and salt stress in terms of synergistic (lower PCD levels), antagonistic (higher PCD levels), or neutral (no net changes in PCD levels) compared to their respective single stress-factor datasets. We organised the unique stress phenotypes displayed by the individual varieties into the form of a stress matrix (**Table 3B**), where most of the stress combination results (75%) fell under the neutral category. Under combined H+S stress, only SW3 and WW4 showed antagonistic interaction, i.e. statistically higher (*p* < 0.05) PCD levels from the H-only control. In contrast, different varieties such as WW1 (synergistic) and WW3 (antagonistic) responded towards S+H treatments. It was intriguing to note that varieties previously singled out as heat and salt tolerant (SW1 and SW2) by their performance at the viability/PCD inflection point (**Table 2**) displayed similar basal tolerance under combined stress exposure as illustrated in **Figure 9**. The inverse situation also held true as individual heat and salt-susceptible varieties (SW4 and WW1) also demonstrated a higher susceptibility to combined stress exposure. **Figure 9** depicts the role of basal tolerance in the correlation between single stress-factor and combined stress exposure; for example, in the S+H dataset, salt-tolerant varieties (SW1 and SW2) had the lowest PCD levels (36-38%), while the salt-susceptible line SW4 had the highest PCD levels (65%). The remaining varieties displayed varying degrees of tolerance: moderately tolerant (WW3 and WW4: 41-42%) and semi-susceptible (SW3, WW1, WW2: 50-54%). A similar scenario was observed in the H+S dataset; thermotolerant SW1 and SW2 varieties had the lowest PCD (24-26%), while the highest PCD levels were seen in SW3, SW4, and WW4 (50-53%). The remaining varieties (WW1, WW2, and WW3) showed varying degrees of tolerance, with PCD ranging from 35 to 43%.

#### Stress-Tolerant Varieties Responded Slower to Priming Compared to Stress-Susceptible Varieties

**Figure 10:**
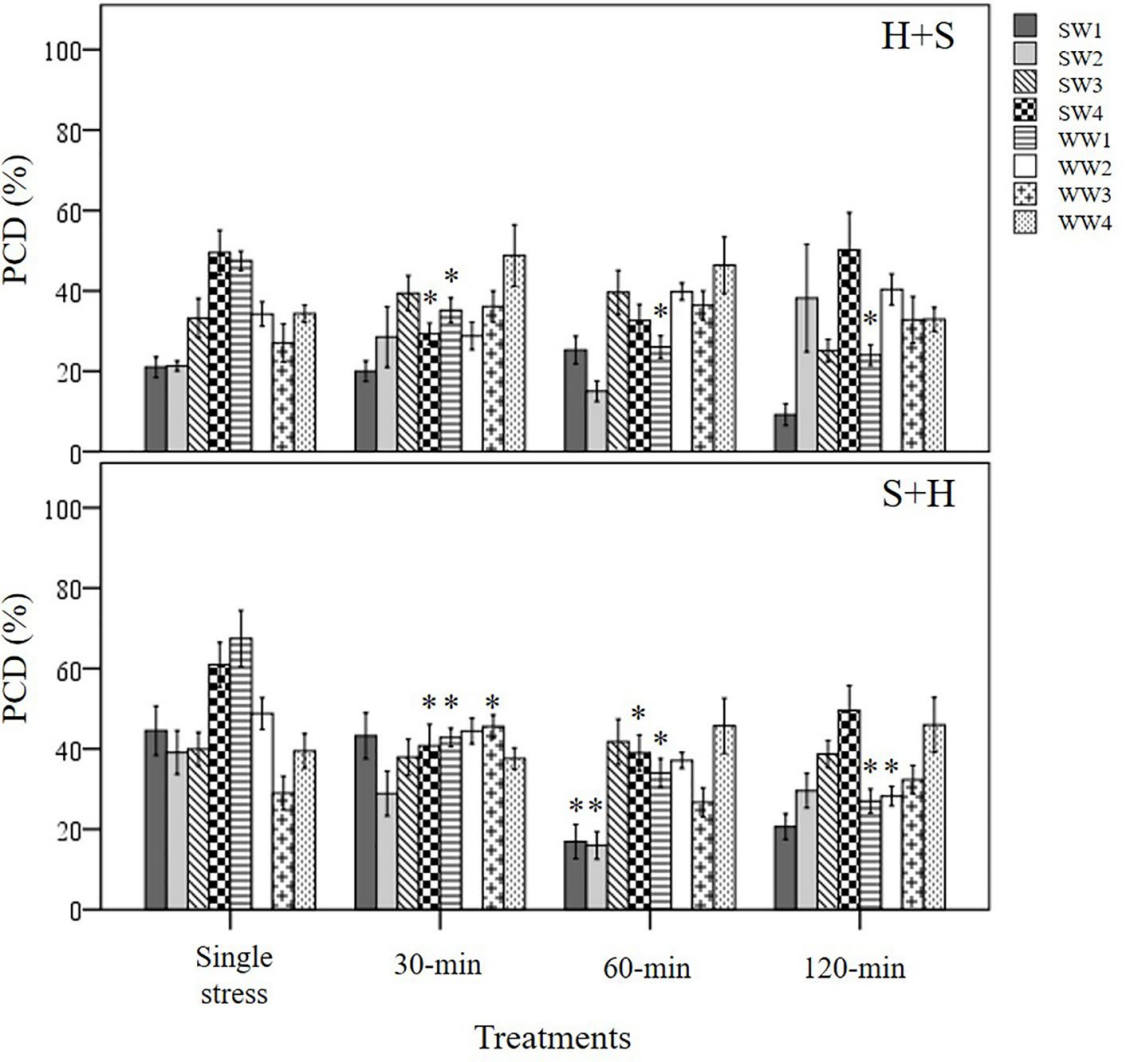
Examining induced tolerance changes across eight individual wheat varieties after different initial stress cues. The initial stress cue (35°C heat or 150 mM NaCl) cue is applied at the 0-min mark, followed by the second stress application at different time-points (30, 60 and 120-min). (H+S) refers to heat stress as the initial cue, followed by salt stress, while (S+H) refers to salt stress as the first cue, followed by heat stress at the relevant time-points. (*) marks PCD results significantly (*p* < 0.05) different from the single-stress factor control (H-only or S-only), using a one-way ANOVA Dunnett post-hoc test (**Supplementary Table 4**). Error bars = standard error of n ≥4 replicates.

Stress-tolerant varieties were predicted to mount a faster counteracting response than stress-susceptible varieties, but this was not evident here. SW1 and SW2 retained similar PCD levels in the 30 min H+S dataset compared to their respective single (H only) stress-factor datasets (**Figure 10**). We only observed cross-stress tolerance to salt stress at the later stages as PCD levels only decreased at the 60-min (SW2) and 120-min (SW1) time-points. In contrast, stress-susceptible SW4 reacted faster as PCD levels declined by 21% (*p* < 0.05) at the 30-min H+S dataset compared to its single heat stress-factor dataset. A similar pattern, although to a lesser extent, appeared in heat primed WW1 seedlings whose PCD levels declined by 12% (*p* < 0.05) at the 30-min dataset compared to the H-only control. In view of the slower adaptive response in stress-tolerant varieties, heat priming enabled the stress susceptible SW4 line to maintain similar PCD levels (28%) in line with the tolerant SW2 variety, despite additional salt stress exposure at the 30-min time-point. Considering how the plant stress response is a combination of both basal and induced tolerance, our results suggests that a rapid induced response can partially make up for low basal tolerance, given successful priming and sufficient time-lag between repeated stresses.

We noted a similar temporal pattern when salt stress was the initial cue; significant cross-stress tolerance (*p* < 0.05) to heat stress only took place at the later stages (60-min) for both stress-tolerant SW1 and SW2 varieties. Like the heat priming treatment, salt priming rapidly suppressed PCD levels in varieties with a low basal tolerance (SW4 and WW1); both lines had statistically lower PCD levels (*p* < 0.05) at the 30-min mark compared to their respective S-only controls. Despite their slower adaptive response, SW1 and SW2 varieties still retained the lowest PCD levels out of all the varieties when the cross-stress tolerant effect finally took place. Regardless of the initial stress cue, PCD levels of the 60-min dataset for SW1 and SW2 initially primed with heat (15-24%) and salt (16-17%), were substantively lower than the average PCD values for the remaining varieties across heat (36.8%) and salt stress (37.5%) priming treatments. On balance, our results show that stress-susceptible varieties responded quicker than stress-tolerant varieties as illustrated in **Figure 10**.

#### Statistical Analysis of the Effect of Using Different Initial Stress Cues on Subsequent PCD Levels

Our results showed that applying salt stress as the initial cue followed by heat stress, exerted a stronger cytotoxic effect on PCD levels (*p*-value = 0.02) compared to the inverse scenario when heat was the first stress cue, despite maintaining identical stress dosages (**Table 4**). Only a small mean difference of 4.3% was observed between the overall (S+H) and (H+S) datasets. However, this only represents the average values across the eight varieties. When controlling for the individual varieties, we saw larger drifts between the H+S and S+H datasets. For example, statistically higher (*p* < 0.05) PCD levels in S+H datasets, compared to H+S datasets were seen in SW1 (11.4%), SW4 (8.5%) and WW1 (5.9%), (**Supplementary Table 8**). It is also worth noting that the stronger PCD-inducing signal in salt-shocked seedlings (S+H) largely disappeared at later time-points, 60 and 120-min. When controlling for PCD levels across the different stress-treatment time-points, the S+H datasets had higher PCD levels than their H+S counterparts at 0-min (S+H: 47.8%; H+S: 40.8%) and 30-min (S+H: 40.6%, H+S: 34.1%), but were similar at the later stages at 60-min (33-34%) and 120-min (33-35%). Hence, the longer the lag between stress applications, the better the priming effect as PCD levels decreased concurrently. One-way ANOVA analysis confirmed this as both the later datasets (60 and 120-min) were significantly lower (*p* < 0.05) than the single salt stress-factor dataset (**Figure 7**). Our results show that given sufficient time, the salt priming effect resulted in similar PCD-suppression rates with seedlings first subjected to heat shock. **Figure 7** illustrates the overall lagging PCD-suppressing effect of the initial salt shock cue, while **Figure 6** shows the how this general behaviour differs from variety to variety.

**Table 4:** Independent samples t-test examining the effects of applying different stress cues as the initial cue on PCD levels. Inputted data consisted of PCD levels scored across 0, 30, 60 and 120-min.

Initial Stress cue	Group Statistics			*t*-test for Equality of Means		
	N	Mean	Std. Error Mean	Sig. (2-tailed)	Mean Difference	Std. Error Difference
Heat and Salt (H+S)	395	35.5	0.964	0.002	-4.31	1.35
Salt and Heat (S+H)	376	39.9	0.947			

## Discussion

In this paper, we present three case studies to illustrate how stress-induced PCD levels can be used to investigate cereal stress tolerance. In the first instance, we directly scored *in vivo* PCD levels in heat-stressed barley and wheat seedlings. We observed mixed thermotolerance across the seasonal wheat varieties but noted a clear distinction between heat resistant spring and heat susceptible winter barley varieties. Without further investigations, it is difficult to determine why these differences exist, as thermotolerance is a spatially and temporally regulated polygenic trait that differs across development stages and plant genotype (Rejeb et al., 2014). However, evidence suggest that heat shock protein (HSP) diversity can be a marker for thermotolerance as Marmiroli et al. (1994) found that low-MW HSP expression patterns differ greatly across five heat-stressed barley varieties of varying thermotolerance. Plant HSPs are molecular chaperones that protect proteins under denaturing conditions and are divided into five families, Hsp100, Hsp90, Hsp70, Hsp60 and small Hsps (sHsp), that occasionally have overlapping functions (Wang et al., 2004). For example, Hsp70 and Hsp90 are engaged in the transcriptional activation of other HSPs, chaperone and stress-response proteins *via* heat-shock factors (HSFs), while sHsp and Hsp70 maintain protein conformation to prevent aggregation (Wang et al., 2004). Genetic HSP diversity might account for the intraspecies variances we noted between the seasonal barley varieties as Marmiroli et al. (1998) found a high degree of polymorphisms at the *Hvhsp17* gene locus that encoded for a low-MW HSP across winter and spring barley varieties. Restriction fragment length polymorphism analysis of two HSP genes (*TaHSP16.9* and *Hvhsp17*) in 27 barley varieties revealed that spring and winter barley varieties could be successfully partitioned into two dendrogram clusters, showing that polymorphisms in the HSP genes accurately predicted winter and spring barley varieties (Marmiroli et al., 1998).

Apart from predicting the divergent thermotolerance between spring and winter barley varieties, HSP molecular diversity might also account for the mixed tolerance of the seasonal wheat varieties we noticed here. Barley plants are diploid organisms, but wheat plants can either have diploid, tetraploid or hexaploid genomes; polyploid cereals have a higher HSP diversity than diploid cereals because of the additive effect of the subgenomes (Maestri et al., 2002). Perhaps this accounts for why mixed tolerance was seen across spring and winter wheat varieties, but not in barley as polyploidy affects HSP diversity and other stress-response genes, culminating in significantly divergent stress phenotypes from their original diploid parents (Arbona et al., 2017).

In the next case study, we examined how stress-induced PCD levels changed across eight salt-stressed wheat varieties. Like earlier heat stress experiments, we noted mixed salt tolerance, which was notably apparent when seedlings were subjected to salt stress at the viable/PCD inflection point. We confirmed the association between heat and salt stress-induced PCD levels with bivariate analysis, showing a statistically significant correlation of 0.333 (*p* < 0.01) between both variables. At this stress dosage, SW1 and SW2 were identified as salt tolerant varieties, SW3, WW3 and WW4 as moderately salt-tolerant, and SW4, WW1 and WW2 as salt susceptible lines. By comparing stress-induced PCD levels at the viable/PCD inflection point, we found striking parallels between heat and salt stress experiments as wheat varieties displayed similar tolerance to heat and salt shock - two seemingly distinct stresses. For example, thermotolerant varieties (SW1 and SW2) retained their robustness to low-to-medium salt stress, while heat susceptible SW4 and WW1 lines were similarly vulnerable to salt stress.

Stress exposure elicits primary and secondary damage (Munns, 2010) and we hypothesise that similar secondary-induced damages was the underlying reason behind the similar tolerance exhibited against heat and salt stress. Plants have evolved stress-specific pathways to deal with initial primary damage, while general ‘housekeeping pathways’ minimise the overlapping secondary damage effects (Munns, 2010). Examples of primary responses include HSP accumulation to counteract the elevated risk of protein misfolding (Wang et al., 2004) while salt-stressed plants upregulate ion transporters for Na^+^ exclusion or sequestration (Wang et al., 2003; Kosová et al., 2015; Kosová et al., 2018). Despite these divergent responses, plants under heat or salt stress will manifest similar secondary damage symptoms in the form of elevated ROS, inhibition of key metabolic enzymes, and macromolecule denaturation (proteins, cell membranes and cytoskeleton) (Rivero et al., 2014). Hence, plants adopt similar downstream protective mechanisms against heat and salt stress because of overlapping secondary damage; examples of shared responses include cell volume regulation (osmolyte and hydrophilic protein accumulation) and upregulation of ROS and methylglyoxal-detoxifying pathways (Hoque et al., 2012; Rivero et al., 2014; Hossain et al., 2016). It is also interesting to note that sHsps are also upregulated during heat, salt and drought stress (Wang et al., 2004; Hossain et al., 2016); sHsps protects the mitochondrial Complex I electron transport chain from oxidative damage in salt-stressed *Z. mays* plants (Hamilton and Heckathorn, 2001), and inhibits PCD by regulating the intracellular redox state in mammalian cells (Arrigo, 1998). Collectively, the evidence suggests that similar tolerance to heat and salt stress by wheat varieties is likely due to higher expression of these conserved response pathways. Our results highlight the flexibility of using stress-induced PCD levels as a general maker for stress tolerance. Like heat tolerance, it is difficult to pinpoint salt tolerance to a single gene as both are polygenic traits controlled by multiple genes and signalling pathways (Zuther et al., 2007; Rejeb et al., 2014). Quantification of PCD levels avoid these problems as it integrates all these interacting networks to yield a useful single end-point measurement of the stress treatment effects. By identifying varieties with stress tolerant traits of interest, further testing using transcriptomics, proteomics and metabolomics can be performed to determine why different lines possess varying degrees of tolerance.

In the final case study, we used stress-induced PCD levels to assess basal, induced and cross-stress tolerance in heat and salt-stressed wheat seedlings. Basal tolerance refers to the innate plant capacity to withstand stress encounters without relying on priming or previous stress exposures, while induced tolerance reflects the adaptive capacity to mount a counteracting response to the initial stress stimuli (Arbona et al., 2017). Unlike genetically pre-determined basal tolerance, induced tolerance can be manipulated by non-lethal stress exposure or priming with chemical modulators for improved stress tolerance (Arbona et al., 2017). Non-lethal stress exposure can lead to improved resistance against additional stress factors, even that of different origins, (i.e. cross-stress tolerance), as the stress imprint can lead to a faster response to recurrent stress-factors compared to plants without a stress memory (Walter et al., 2013). But if the initial stress-factor undermines the plant defence or irreversibly disrupts cellular homeostasis, repeated stress exposure leads to an even greater harm (Walter et al., 2013). Therefore, depending on the adaptability of the induced tolerance response, the second stress application can either have a net positive, negative, or neutral effect on the plant stress response (**Supplementary Figure 1B**).

As illustrated in **Figure 1**, we assessed basal tolerance by subjecting seedlings to single and combined stress exposures, while induced and cross-stress tolerance were examined by applying recurrent stress cues of different origins. We subjected seedlings to different stress combinations because while individual stress exposures have been intensely researched over the years, plants continually experience unique stress combinations under field conditions (Mittler, 2006). For example, farmlands in the semi-arid regions of the world tend to face a combination of salt, heat and drought stress (Rivero et al., 2014). Evidence indicates that plants under combined stress display a unique ‘stress phenotype’ that has little overlap with the phenotype exhibited under individual stresses. Hence, there are growing calls to study how plants respond under conditions that mimic field conditions, as the novel stress response under two different combined stresses cannot be merely extrapolated from studies where stresses were applied individually (Rasmussen et al., 2013; Rivero et al., 2014). This was demonstrated in a landmark study by Rasmussen et al. (2013) who discovered that 61% of the transcripts from double-combined stress exposure could not be anticipated from their individual stress treatments alone.

The survival of plants against stress depends on basal and adaptive tolerance and we noted a few interesting observations when screening seedlings for these attributes. First, varieties (SW1 and SW2) previously singled out as tolerant to single heat and salt stress exposure also exhibited similar resistance to combined and multiple individual stresses. In the case of SW1 and SW2, basal tolerance likely played a bigger initial role as both varieties had the lowest stress-induced PCD levels upon combined stress exposure, which did not significantly change from their respective single (heat or salt) stress-factor control. Thus, the single and combined stress-factor datasets strongly suggest that SW1 and SW2 have inherently high basal tolerance compared to the other varieties.

Cross-stress tolerance is the phenomenon where the initial stress exposure makes plants more resistant to other stress types, and SW1 and SW2 had an unexpectedly slower cross-tolerance response than their stress-susceptible counterparts. Both lines were initially hypothesised to have a rapidly induced tolerance response as Kawasaki et al. (2001) previously showed that salt-tolerant rice (Pokkali) responded faster to salt stress than the salt-sensitive (IR29) line. Transcription upregulation in Pokkali started a mere 15 min after the shock, while IR29 had a four-fold delayed response, suggesting that its slow ability to process stress cues was the underlying reason for its ineffective salt stress response (Kawasaki et al., 2001). However, we did not observe any significant changes in overall PCD levels in SW1 and SW2 when the secondary stress cue was applied at 30-min. Instead, the beneficial PCD-suppressing effects were only noted when the stress cue was applied at the later stages. This stands in contrast to stress-susceptible varieties with low basal tolerance, like SW4 and WW1, that adapted faster to recurrent stresses. Both lines had substantially lower PCD levels, even when the second stress cue was applied at the 30-min time-point, showing that the first non-lethal stress successfully primed SW4 and WW1 against additive damage from recurrent stress exposure. Collectively, our results show that stress-susceptible varieties responded faster than stress-tolerant varieties and evidence suggest that signalling components play a prominent role in this process as they control the reprogramming of cellular molecular machinery (Rejeb et al., 2014). For example, the transcriptional regulator MBF1c modulates basal thermotolerance but not induced tolerance (Ahammed et al., 2016), ABA-deficient *Arabidopsis* mutants had substantial losses of basal and acquired thermotolerance (Larkindale et al., 2005), while salicylic acid-dependent signalling increases basal thermotolerance but not induced tolerance (Clarke et al., 2004). Other studies have also shown that the signalling molecules ROS and methylglyoxal successfully imprinted cross-stress tolerance against drought and salt stress in *Brassica campestris* L. (Hossain et al., 2013; Hossain et al., 2016), while mechanical wounding increased salt tolerance in tomato plants because of cross-talk between the signalling pathways involving calmodulin-like activities, the signalling peptide systemin, and jasmonic acid biosynthesis (Capiati et al., 2006). Further work will be needed to deduce the role of these signalling molecules in the identified varieties of interest, but our results show that stress-induced PCD levels can be a useful marker of ecological stress memory. The identified stress-susceptible varieties had faster induced tolerance; despite the short time-lag between the two stress applications, both lines had not returned to their earlier homeostatic state and mounted a faster counteracting response and were consequently more tolerant against repeated stress - even that of a different origin (Walter et al., 2013).

It is also worth noting that the favoured modes of stress-response employed by stress-tolerant SW1 and SW2 (high basal tolerance, but slow induced response) and stress-susceptible SW4 and WW1 (low basal tolerance, but fast induced response) is remarkably similar to the strategies employed by two species of poplar tree: salt tolerant *Populus euphratica* and salt susceptible *Populus* × *canescens* (Janz et al., 2010). The elevated basal tolerance of *P. euphratica* was reflected in the high constitutive expression of salt sensitive genes but had comparatively low transcriptional responsiveness compared to *P*. x *canescens*. Salt-tolerant *P. euphratica* was slower to react to external changes in salt levels and did not rely on a global defence strategy unlike its salt-susceptible counterpart. Instead, *P. euphratica* were already pre-adapted to osmotic stress by the constitutive activation of cell protective mechanisms involved in ROS detoxification, osmolyte biosynthesis, Na^+^ and K^+^ ion carriers, and metabolite transporters. However, permanent activation of these pathways imposed a high metabolic burden and Janz et al. (2010) suggested this stress-anticipatory preparedness comes at the expense of diminished flexibility and a slower transcriptome response against fluctuating salt levels. Perhaps a comparable scenario is at play for SW1 and SW2 given that despite their slower induced tolerance, both varieties had the lowest overall PCD levels because of their inherently high basal tolerance.

Last, we observed an interesting phenomenon when different initial stress cues were used during combined and multiple individual stress experiments. Plants display a unique stress phenotype under combined stress that has little overlap with individual stress treatments (Mittler, 2006; Rasmussen et al., 2013; Rivero et al., 2014). Sometimes, combined stress can result in better plant robustness, e.g. mechanical injury increased salt tolerance of tomato plants (Capiati et al., 2006) or elevated vulnerability to the second stress, e.g. heavy metal exposure aggravated the effects of drought stress (Barceló and Poschenrieder, 1990). Based on the stress phenotype categories devised by Mittler (2006), we observed the dominance of salt stress over heat stress under combined stress exposure. Even though identical stress doses were maintained, salt stress application followed by subsequent heat stress, exerted a stronger cytotoxic effect on PCD levels compared to the reverse scenario. Our results align with past *Arabidopsis* transcriptomic data showing that plants under combined stress prioritizes the salt-stress response over heat; Rasmussen et al. (2013) found that heat and salt-stressed plants had the highest level of prioritized transcripts (12.1%) out of six stress combinations. The greater response of salt transcripts compared to heat transcripts showed that the salt response dominated the heat stress response (Rasmussen et al., 2013). Taken together, our results with stress-induced PCD levels also accurately depicted the dominance of salt stress over heat stress, as shown in past transcriptomic data (Rasmussen et al., 2013). Nevertheless, we wish to reiterate that this finding simply reflects the overall trends as the individual stress response can vary between the varieties, as shown in the stress matrix (**Table 3**). Most varieties responded neutrally to combined heat and salt stress, although there were a few outliers for antagonistic (SW3, WW3, and WW4) and synergistic (WW1) interactions. Our results concur with past observations that plants display a unique stress phenotype when subjected to overlapping stress that is not necessarily additive, and that combined stress should be regarded as a new state of abiotic stress that requires a novel adaptive stress response (Mittler, 2006; Rasmussen et al., 2013; Rivero et al., 2014). Finally, we would like to extend an important caveat to the original hypothesis, as our results show that stress phenotypes can vary even within different varieties of the same species, and that caution should be exercised when extrapolating findings across different research groups. This intra-species diversity can be advantageous as the RHA enables agronomists to identify stress-tolerant varieties early in the screening process, without relying on exhaustive large-scale field trials or costly analytical chemistry and molecular biology techniques.

## Conclusion

This paper demonstrates the use of root hairs as a model system for studying plant stress tolerance as direct scoring of stress-induced PCD levels integrates multiple stress-response pathways for a simple outcome, i.e. do plant cells stay alive or undergo PCD. A graphical summary of the findings obtained by the study is shown in **Supplementary Figure 3**. The RHA was originally developed in *Arabidopsis* and, in this study, the method was successfully applied on cereals to evaluate the heat and/or salt tolerance of barley and wheat varieties. By examining heat stress-induced PCD levels, a clear distinction between thermotolerant spring and thermo-susceptible winter barley varieties was determined. In addition, eight wheat varieties were examined for their tolerance to heat and salt stress; a comparison of their individual viability/PCD inflection points identified stress tolerant (SW1 and SW2) and stress susceptible (SW4 and WW1) varieties. Following this finding, stress-induced PCD levels were used to assess the basal, induced and cross-stress tolerance of the eight wheat varieties to heat and salt stress using single, multiple individual and combined stress exposures, respectively. Interesting parallels could be drawn from the earlier single-stress experiments as the same varieties demonstrated similar cross-stress tolerance (SW1 and SW2) and susceptibility (SW4 and WW1) to heat and salt stress.

Our results also show that stress-tolerant varieties (SW1 and SW2) had high basal tolerance, but a slower induced response compared to stress-susceptible varieties (SW4 and WW1). In addition, the dominant, more damaging effect of salt over heat stress was demonstrated; application of salt stress as the first stress cue induced a stronger cytotoxic effect than heat stress even though identical stress doses were maintained. The strength of the RHA lies in its simplicity and scalability as it can be easily adapted across various plant species and stress protocols in a simple ‘plug-and-play’ fashion. Last, we show that stress-induced PCD levels can be used for identifying cereal varieties with notable stress-tolerance traits for downstream work and for investigaing unique stress-phenotypes exhibited under combined stress, all in a fast and economical manner.

## Data Availability Statement

All datasets generated for this study are included in the article/**Supplementary Material**.

## Author Contributions

AC designed and performed the experiments. LF and CD contributed to the discussion of the results. All authors reviewed and approved the final manuscript.

## Funding

This work was supported by PhD scholarship awarded to AC by Waterford Institute of Technology. Cost of publishing was supported by the Waterford Institute of Technology, School of Science and Computing, Research Support Scheme.

## Conflict of Interest

The authors declare that the research was conducted in the absence of any commercial or financial relationships that could be construed as a potential conflict of interest.

## References

[B1] Ahammed G. J. Li X. Zhou J. Zhou Y.-H. Yu J.-Q. (2016). “Role of hormones in plant adaptation to heat stress,” in Plant Hormones under Challenging Environmental Factors. Eds. Ahammed G. J. Yu J.-Q. (Dordrecht: Springer Netherlands), 1–21. 10.1007/978-94-017-7758-2_1

[B2] Ahmad P. Abdel Latef A. A. H. Rasool S. Akram N. A. Ashraf M. Gucel S. (2016). Role of proteomics in crop stress tolerance. Front. Plant Sci. 7, 1336. 10.3389/fpls.2016.01336 27660631 PMC5014855

[B3] Arbona V. Manzi M. Zandalinas S. I. Vives-Peris V. Pérez-Clemente R. M. Gómez-Cadenas A. (2017). “Physiological, metabolic, and molecular responses of plants to abiotic stress,” in Stress Signaling in Plants: Genomics and Proteomics Perspective, Volume 2. Eds. Sarwat M. Ahmad A. Abdin M. Z. Ibrahim M. M. (Cham: Springer International Publishing), 1–35. 10.1007/978-3-319-42183-4_1

[B4] Arrigo A. P. (1998). Small stress proteins: chaperones that act as regulators of intracellular redox state and programmed cell death. Biol. Chem. 379, 19–26. 10.1007/978-3-642-56348-5_9 9504712

[B5] Barceló J. Poschenrieder C. (1990). Plant water relations as affected by heavy metal stress: a review. J. Plant Nutr. 13, 1–37. 10.1080/01904169009364057

[B6] Capiati D. A. País S. M. Téllez-Iñón M. T. (2006). Wounding increases salt tolerance in tomato plants: evidence on the participation of calmodulin-like activities in cross-tolerance signalling. J. Exp. Bot. 57, 2391–2400. 10.1093/jxb/erj212 16766597

[B7] Cattivelli L. Baldi P. Crosatti C. Di Fonzo N. Faccioli P. Grossi M. (2002). Chromosome regions and stress-related sequences involved in resistance to abiotic stress in *Triticeae*. Plant Mol. Biol. 48, 649–665. 10.1023/A:1014824404623 11999841

[B8] Chen J. Bellin D. Vandelle E. (2018). “Measurement of cyclic GMP during plant hypersensitive disease resistance response,” in Plant Programmed Cell Death. Eds. De Gara L. Locato V. (New York, NY: Springer New York), 143–151. 10.1007/978-1-4939-7668-3_13 29332293

[B9] Chinnusamy V. Zhu J.-K. Sunkar R. (2010). “Gene regulation during cold stress acclimation in plants,” in Plant Stress Tolerance. Ed. Sunkar R. (Totowa, NJ: Humana Press), 39–55. 10.1007/978-1-60761-702-0_3 PMC3064467 20387039

[B10] Cimini S. Ronci M. B. Barizza E. de Pinto M. C. Locato V. Lo Schiavo F. (2018). “Plant cell cultures as model systems to study programmed cell death,” in Plant Programmed Cell Death. Eds. De Gara L. Locato V. (New York, NY: Springer New York), 173–186. 10.1007/978-1-4939-7668-3_16 29332296

[B11] Clarke S. M. Mur L. A. J. Wood J. E. Scott I. M. (2004). Salicylic acid dependent signaling promotes basal thermotolerance but is not essential for acquired thermotolerance in *Arabidopsis thaliana*. Plant J. 38, 432–447. 10.1111/j.1365-313X.2004.02054.x 15086804

[B12] Cockram J. Chiapparino E. Taylor S. A. Stamati K. Donini P. Laurie D. A. (2007). Haplotype analysis of vernalization loci in European barley germplasm reveals novel *VRN-H1* alleles and a predominant winter *VRN-H1/VRN-H2* multi-locus haplotype. Theor. Appl. Genet. 115, 993–1001. 10.1007/s00122-007-0626-x 17713756

[B13] Coleman-Derr D. Tringe S. G. (2014). Building the crops of tomorrow: Advantages of symbiont-based approaches to improving abiotic stress tolerance. Front. In Microbiol. 5, 1–6. 10.3389/fmicb.2014.00283 PMC4047557 24936202

[B14] Distelfeld A. Li C. Dubcovsky J. (2009). Regulation of flowering in temperate cereals. Curr. Opin. In Plant Biol. 12, 178–184. 10.1016/j.pbi.2008.12.010 19195924

[B15] Doccula F. G. Luoni L. Behera S. Bonza M. C. Costa A. (2018). “*In vivo* analysis of calcium levels and glutathione redox status in *Arabidopsis* epidermal leaf cells infected with the hypersensitive response-inducing bacteria *Pseudomonas syringae* pv. tomato *Avrb* (*PstAvrB*),” in Plant Programmed Cell Death. Eds. De Gara L. Locato V. (New York, NY: Springer New York), 125–141. 10.1007/978-1-4939-7668-3_12 29332292

[B16] Elavarthi S. Martin B. (2010). “Spectrophotometric assays for antioxidant enzymes in plants,” in Plant Stress Tolerance. Ed. Sunkar R. (Totowa, NJ: Humana Press), 273–280. 10.1007/978-1-60761-702-0_16 20387052

[B17] Hamilton E. W. Heckathorn S. A. (2001). Mitochondrial adaptations to NaCl. Complex I is protected by anti-oxidants and small heat shock proteins, whereas Complex II is protected by proline and betaine. Plant Physiol. 126, 1266–1274. 10.1104/pp.126.3.1266 11457977 PMC116483

[B18] Hatsugai N. Hara-Nishimura I. (2018). “Measurement of the caspase-1-like activity of vacuolar processing enzyme in plants,” in Plant Programmed Cell Death. Eds. De Gara L. Locato V. (New York, NY: Springer New York), 163–171. 10.1007/978-1-4939-7668-3_15 29332295

[B19] Hoang T. M. L. Williams B. Mundree S. G. (2016). “Manipulation of programmed cell death pathways enhances osmotic stress tolerance in plants: physiological and molecular insights,” in Drought Stress Tolerance in Plants, vol. 1 Eds. Hossain M. A. Wani S. H. Bhattacharjee S. Burritt D. J. Tran L.-S. P. (Cham: Springer International Publishing), 439–464. 10.1007/978-3-319-28899-4_19

[B20] Hogg B. V. Kacprzyk J. Molony E. M. Reilly C. O. Gallagher T. F. Gallois P. (2011). An *in vivo* root hair assay for determining rates of apoptotic-like programmed cell death in plants. Plant Methods 7, 45. 10.1186/1746-4811-7-45 22165954 PMC3266644

[B21] Hoque M. A. Uraji M. Torii A. Banu M. Akhter N. Mori I. C. (2012). Methylglyoxal inhibition of cytosolic ascorbate peroxidase from *Nicotiana tabacum*. J. Biochem. Mol. Toxicol. 26, 315–321. 10.1002/jbt.21423 22696433

[B22] Hossain M. A. Golam Mostofa M. Fujita M. (2013). Heat-shock positively modulates oxidative protection of salt and drought-stressed mustard (*Brassica campestris* L.) seedlings. J. Plant Sci. Mol. Breed. 2, 2. 10.7243/2050-2389-2-2

[B23] Hossain M. A. Burritt D. J. Fujita M. (2016). “Cross-stress tolerance in plants: Molecular mechanisms and possible involvement of reactive oxygen species and methylglyoxal detoxification systems,” in Abiotic Stress Response in Plants (John Wiley & Sons, Ltd) (Weinheim, Germany: Wiley), 327–380. 10.1002/9783527694570.ch16

[B24] Jambunathan N. (2010). “Determination and detection of reactive oxygen species (ROS), lipid peroxidation, and electrolyte leakage in plants,” in Plant Stress Tolerance Methods in Molecular Biology. Ed. Sunkar R. (Totowa, NJ: Humana Press), 291–297. 10.1007/978-1-60761-702-0_18 20387054

[B25] Janz D. Behnke K. Schnitzler J.-P. Kanawati B. Schmitt-Kopplin P. Polle A. (2010). Pathway analysis of the transcriptome and metabolome of salt sensitive and tolerant poplar species reveals evolutionary adaption of stress tolerance mechanisms. BMC Plant Biol. 10, 150. 10.1186/1471-2229-10-150 20637123 PMC3095294

[B26] Kacprzyk J. Daly C. T. McCabe P. F. (2011). “The botanical dance of death: Programmed cell death in plants,” in Advances in Botanical Research (San Diego, US: Elsevier), 169–261. 10.1016/B978-0-12-385851-1.00004-4

[B27] Kacprzyk J. Devine A. McCabe P. F. (2014). The root hair assay facilitates the use of genetic and pharmacological tools in order to dissect multiple signalling pathways that lead to programmed cell death. PloS One 9, e94898. 10.1371/journal.pone.0094898 24755572 PMC3995694

[B28] Kacprzyk J. Brogan N. P. Daly C. T. Doyle S. M. Diamond M. Molony E. M. (2017). The retraction of the protoplast during PCD is an active, and interruptible, calcium-flux driven process. Plant Sci. 260, 50–59. 10.1016/j.plantsci.2017.04.001 28554474

[B29] Kawasaki S. Borchert C. Deyholos M. Wang H. Brazille S. Kawai K. (2001). Gene expression profiles during the initial phase of salt stress in rice. Plant Cell 13, 889. 10.1105/tpc.13.4.889 11283343 PMC135538

[B30] Kim Y. Wang M. Bai Y. Zeng Z. Guo F. Han N. (2014). Bcl-2 suppresses activation of VPEs by inhibiting cytosolic Ca^2+^ level with elevated K^+^ efflux in NaCl-induced PCD in rice. Plant Physiol. Biochem. 80, 168–175. 10.1016/j.plaphy.2014.04.002 24787501

[B31] Kosová K. Vítámvás P. Urban M. Klíma M. Roy A. Prášil I. (2015). Biological networks underlying abiotic stress tolerance in temperate crops—A proteomic perspective. Int. J. Mol. Sci. 16, 20913–20942. 10.3390/ijms160920913 26340626 PMC4613235

[B32] Kosová K. Vítámvás P. Urban M. O. Prášil I. T. Renaut J. (2018). Plant abiotic stress proteomics: The major factors determining alterations in cellular proteome. Front. In Plant Sci. 9, 122. 10.3389/fpls.2018.00122 29472941 PMC5810178

[B33] Larkindale J. Hall J. D. Knight M. R. Vierling E. (2005). Heat stress phenotypes of *Arabidopsis* mutants implicate multiple signaling pathways in the acquisition of thermotolerance. Plant Physiol. 138, 882–897. 10.1104/pp.105.062257 15923322 PMC1150405

[B34] Lockshin R. A. Zakeri Z. (2004). Apoptosis, autophagy, and more. Int. J. Biochem. Cell Biol. 36, 2405–2419. 10.1016/j.biocel.2004.04.011 15325581

[B35] Maestri E. Klueva N. Perrotta C. Gulli M. Nguyen H. T. Marmiroli N. (2002). Molecular genetics of heat tolerance and heat shock proteins in cereals. Plant Mol. Biol. 48, 667–681. 10.1023/A 11999842

[B36] Mano J. Biswas M. S. (2018). “Analysis of reactive carbonyl species generated under oxidative stress,” in Plant Programmed Cell Death. Eds. De Gara L. Locato V. (New York, NY: Springer New York), 117–124. 10.1007/978-1-4939-7668-3_11 29332291

[B37] Marmiroli N. Maestri E. Terzi V. Gulli M. Pavesi A. Raho G. (1994). “Genetic and molecular evidences of the regulation of gene expression during heat shock in plants,” in Biochemical and Cellular Mechanisms of Stress Tolerance in Plants NATO ASI Series. Ed. Cherry J. H. (Berlin, Germany: Springer), 157–190.

[B38] Marmiroli N. Malcevschi A. Maestri E. (1998). “Application of stress responsive genes RFLP analysis to the evaluation of genetic diversity in plants,” in Molecular Tools for Screening Biodiversity: Plants and Animals. Eds. Karp A. Isaac P. G. Ingram D. S. (Dordrecht: Springer Netherlands), 464–470. 10.1007/978-94-009-0019-6_82

[B39] Meena K. K. Sorty A. M. Bitla U. M. Choudhary K. Gupta P. Pareek A. (2017). Abiotic stress responses and microbe-mediated mitigation in plants: the omics strategies. Front. In Plant Sci. 8, 172. 10.3389/fpls.2017.00172 28232845 PMC5299014

[B40] Mittler R. Blumwald E. (2010). Genetic engineering for modern agriculture: challenges and perspectives. Annu. Rev. Plant Biol. 61, 443–462. 10.1146/annurev-arplant-042809-112116 20192746

[B41] Mittler R. (2006). Abiotic stress, the field environment and stress combination. Trends In Plant Sci. 11, 15–19. 10.1016/j.tplants.2005.11.002 16359910

[B42] Munns R. Wallace P. A. Teakle N. L. Colmer T. D. (2010). “Measuring soluble ion concentrations (Na^+^, K^+^, Cl^–^) in salt-treated plants,” in Plant Stress Tolerance. Ed. Sunkar R. (Totowa, NJ: Humana Press), 371–382. 10.1007/978-1-60761-702-0_23 20387059

[B43] Munns R. (2010). Approaches to identifying genes for salinity tolerance and the importance of timescale. Methods Mol. Biol. 639, 25–38. 10.1007/978-1-60761-702-0_2 20387038

[B44] Obata T. Fernie A. R. (2012). The use of metabolomics to dissect plant responses to abiotic stresses. Cell. Mol. Life Sci. 69, 3225–3243. 10.1007/s00018-012-1091-5 22885821 PMC3437017

[B45] Pandey P. Irulappan V. Bagavathiannan M. V. Senthil-Kumar M. (2017). Impact of combined abiotic and biotic stresses on plant growth and avenues for crop improvement by exploiting physio-morphological traits. Front. Plant Sci. 8, 537. 10.3389/fpls.2017.00537 28458674 PMC5394115

[B46] Petrov V. Hille J. Mueller-Roeber B. Gechev T. S. (2015). ROS-mediated abiotic stress-induced programmed cell death in plants. Front. Plant Sci. 6, 1–16. 10.3389/fpls.2015.00069 25741354 PMC4332301

[B47] Rasmussen S. Barah P. Suarez-Rodriguez M. C. Bressendorff S. Friis P. Costantino P. (2013). Transcriptome responses to combinations of stresses in *Arabidopsis*. Plant Physiol. 161, 1783–1794. 10.1104/pp.112.210773 23447525 PMC3613455

[B48] Reape T. J. Brogan N. P. McCabe P. F. (2015). “Mitochondrion and chloroplast regulation of plant programmed cell death,” in Plant Programmed Cell Death. Eds. Gunawardena A. N. McCabe P. F. (Cham: Springer International Publishing), 33–53. 10.1007/978-3-319-21033-9_2

[B49] Rejeb I. Pastor V. Mauch-Mani B. (2014). Plant responses to simultaneous biotic and abiotic stress: molecular mechanisms. Plants 3, 458–475. 10.3390/plants3040458 27135514 PMC4844285

[B50] Rivero R. M. Mestre T. C. Mittler R. Rubio F. Garcia-Sanchez F. Martinez V. (2014). The combined effect of salinity and heat reveals a specific physiological, biochemical and molecular response in tomato plants: Stress combination in tomato plants. Plant Cell Environ. 37, 1059–1073. 10.1111/pce.12199 24028172

[B51] Singh B. Mishra S. Bohra A. Joshi R. Siddique K. H. M. (2018). “Crop phenomics for abiotic stress tolerance in crop plants,” in Biochemical, Physiological and Molecular Avenues for Combating Abiotic Stress Tolerance in Plants. Ed. Wani S. H. (San Diego, US: Elsevier), 277–296. 10.1016/B978-0-12-813066-7.00015-2

[B52] Tausz M. Šircelj H. Grill D. (2004). The glutathione system as a stress marker in plant ecophysiology: Is a stress-response concept valid? J. Exp. Bot. 55, 1955–1962. 10.1093/jxb/erh194 15234995

[B53] Terrón-Camero L. C. Molina-Moya E. Sanz-Fernández M. Sandalio L. M. Romero-Puertas M. C. (2018). “Detection of reactive oxygen and nitrogen species (ROS/RNS) during hypersensitive cell death,” in Plant Programmed Cell Death. Eds. De Gara L. Locato V. (New York, NY: Springer New York), 97–105. 10.1007/978-1-4939-7668-3_9 29332289

[B54] United Nations, Department of Economic and Social Affairs, Population Division (2017). World Population Prospects: The 2017 Revision, Key Findings and Advance Tables. Working Paper No. ESA/P/WP/248. Accessed 19th November 2019 at: https://population.un.org/wpp/Publications/Files/WPP2017_KeyFindings.pdf

[B55] Verslues P. E. (2010). “Quantification of water stress-induced osmotic adjustment and proline accumulation for *Arabidopsis thaliana* molecular genetic studies,” in Plant Stress Tolerance Methods in Molecular Biology. Ed. Sunkar R. (Totowa, NJ: Humana Press), 301–315. 10.1007/978-1-60761-702-0_19 20387055

[B56] Walter J. Jentsch A. Beierkuhnlein C. Kreyling J. (2013). Ecological stress memory and cross stress tolerance in plants in the face of climate extremes. Environ. Exp. Bot. 94, 3–8. 10.1016/j.envexpbot.2012.02.009

[B57] Wang W. Vinocur B. Altman A. (2003). Plant responses to drought, salinity and extreme temperatures: towards genetic engineering for stress tolerance. Planta 218, 1–14. 10.1007/s00425-003-1105-5 14513379

[B58] Wang W. Vinocur B. Shoseyov O. Altman A. (2004). Role of plant heat-shock proteins and molecular chaperones in the abiotic stress response. Trends In Plant Sci. 9, 244–252. 10.1016/j.tplants.2004.03.006 15130550

[B59] Wituszynska W. Karpinski S. (2013). “Programmed cell death as a response to high light, UV and drought stress in plants,” in Abiotic Stress - Plant Responses and Applications in Agriculture. Ed. Vahdati K. (London, UK: InTech). 10.5772/53127

[B60] Wu Q. Jackson D. (2018). “Detection of MAPK3/6 phosphorylation during hypersensitive response (HR)-associated programmed cell death in plants,” in Plant Programmed Cell Death. Eds. De Gara L. Locato V. (New York, NY: Springer New York), 153–161). 10.1007/978-1-4939-7668-3_14 29332294

[B61] Xiao D. He H. Huang W. Oo T. L. Wang A. He L.-F. (2018). Analysis of mitochondrial markers of programmed cell death. Methods Mol. Biol. 1743, 65–71. 10.1007/978-1-4939-7668-3_6 29332286

[B62] Zuther E. Koehl K. Kopka J. (2007). “Comparative metabolome analysis of the salt response in breeding cultivars of rice,” in Advances in Molecular Breeding Toward Drought and Salt Tolerant Crops. Eds. Jenks M. A. Hasegawa P. M. Jain S. M. (Dordrecht: Springer Netherlands), 285–315. 10.1007/978-1-4020-5578-2_12

