# Supplementary material for: Sorting the Wheat From the Chaff: Programmed Cell Death as a Marker of Stress Tolerance in Agriculturally Important Cereals
Source: Front Plant Sci. 2019 Nov 26;10:1539. doi: 10.3389/fpls.2019.01539 (PMC6888703; doi:10.3389/fpls.2019.01539)
Supplement: Supplementary file 1 [file DataSheet_1.docx]

Supplementary Material: Sorting the wheat from the chaff: PCD as a marker of stress tolerance in agriculturally important cereals

Supplementary Table 1 – Effect of heat stress on PCD levels in winter (WB) and spring (SB) barley varieties. One-way ANOVA (Dunnett post-hoc) was used for statistical analysis which treated the 25 °C dataset as a control and compared all other group datasets against it. (*) indicates the mean difference is significant at the 0.05 level. Error bars = standard error of n ≥ 8 replicates.

| **Barley Variety** | **Temperature (°C)** | **% PCD** | **Mean difference from 25 °C dataset (%)** | ***p*-value** |
| --- | --- | --- | --- | --- |
| **WB1** | 25 | 43.38 ± 3.73 | N/A | N/A |
|  | 35 | 86.47 ± 3.34 | 43.1* | 0.000 |
|  | 45 | 86.90 ± 3.20 | 43.5* | 0.000 |
|  | 50 | 97.84 ± 0.61 | 54.5* | 0.000 |
|  | 55 | 96.46 ± 1.38 | 53.1* | 0.000 |
| **WB2** | 25 | 34.93 ± 4.85 | N/A | N/A |
|  | 35 | 63.40 ± 5.37 | 28.5* | 0.000 |
|  | 45 | 83.88 ± 5.14 | 48.9* | 0.000 |
|  | 50 | 97.85 ± 0.53 | 62.9* | 0.000 |
|  | 55 | 97.55 ± 0.56 | 62.6* | 0.000 |
| **WB3** | 25 | 39.94 ± 5.78 | N/A | N/A |
|  | 35 | 42.92 ± 9.23 | 3.0 | 0.987 |
|  | 45 | 82.53 ± 4.44 | 42.6* | 0.000 |
|  | 50 | 97.21 ± 0.46 | 57.3* | 0.000 |
|  | 55 | 91.11 ± 2.09 | 51.2* | 0.000 |
| **WB4** | 25 | 35.96 ± 3.62 | N/A | N/A |
|  | 35 | 55.09 ± 5.45 | 19.1* | 0.001 |
|  | 45 | 83.88 ± 4.07 | 47.9* | 0.000 |
|  | 50 | 97.03 ± 0.73 | 61.1* | 0.000 |
|  | 55 | 95.77 ± 0.83 | 59.8* | 0.000 |
| **SB1** | 25 | 10.21 ± 1.58 | N/A | N/A |
|  | 35 | 12.94 ± 1.85 | 2.7 | 0.906 |
|  | 45 | 68.62 ± 5.06 | 58.4* | 0.000 |
|  | 50 | 93.40 ± 2.31 | 83.2* | 0.000 |
|  | 55 | 93.47 ± 1.36 | 83.3* | 0.000 |
| **SB2** | 25 | 14.35 ± 2.98 | N/A | N/A |
|  | 35 | 17.22 ± 2.45 | 2.9 | 0.834 |
|  | 45 | 65.44 ± 3.33 | 51.1* | 0.000 |
|  | 50 | 95.01 ± 0.96 | 80.7* | 0.000 |
|  | 55 | 91.92 ± 1.43 | 77.6* | 0.000 |
| **SB3** | 25 | 11.27 ± 2.43 | N/A | N/A |
|  | 35 | 10.04 ± 1.81 | -1.2 | 0.999 |
|  | 45 | 56.56 ± 6.66 | 45.3* | 0.000 |
|  | 50 | 96.03 ± 1.85 | 84.8* | 0.000 |
|  | 55 | 87.06 ± 2.81 | 75.8* | 0.000 |

Supplementary Table 2 - Effect of heat stress PCD levels in spring (SW) and winter (WW) wheat varieties. One-way ANOVA (Dunnett post-hoc) was used for statistical analysis which treated the 25 °C dataset as a control and compared all other group datasets against it. (*) The mean difference is significant at the 0.05 level. Error bars = standard error of n ≥ 12 replicates.

| **Wheat Variety** | **Temperature (°C)** | **% PCD** | **Mean difference from 25 °C dataset (%)** | ***p*-value** |
| --- | --- | --- | --- | --- |
| **SW1** | 25 | 9.10 ± 2.36 | N/A | N/A |
|  | 35 | 17.3 ± 2.94 | 8.200 | 0.229 |
|  | 45 | 63.8 ± 4.70 | 54.7 * | 0.000 |
|  | 50 | 91.3 ± 1.58 | 82.2 * | 0.000 |
|  | 55 | 92.7 ± 1.67 | 83.6 * | 0.000 |
| **SW2** | 25 | 11.9 ± 3.06 | N/A | N/A |
|  | 35 | 20.7 ± 1.80 | 8.89 | 0.085 |
|  | 45 | 71.5 ± 4.86 | 59.6 * | 0.000 |
|  | 50 | 90.9 ± 1.39 | 79.0 * | 0.000 |
|  | 55 | 90.1 ± 1.46 | 78.3 * | 0.000 |
| **SW3** | 25 | 23.9 ± 1.90 | N/A | N/A |
|  | 35 | 28.0 ± 2.64 | 4.1 | 0.482 |
|  | 45 | 86.9 ± 3.03 | 63.0 * | 0.000 |
|  | 50 | 91.5 ± 0.84 | 67.7 * | 0.000 |
|  | 55 | 92.8 ± 0.94 | 69.0 * | 0.000 |
| **SW4** | 25 | 36.8 ± 4.20 | N/A | N/A |
|  | 35 | 46.5 ± 6.14 | 9.63 | 0.238 |
|  | 45 | 88.3 ± 2.92 | 51.5 * | 0.000 |
|  | 50 | 93.0 ± 1.35 | 56.2 * | 0.000 |
|  | 55 | 94.1 ± 0.55 | 57.3 * | 0.000 |
| **WW1** | 25 | 53.2 ± 3.80 | N/A | N/A |
|  | 35 | 47.4 ± 3.58 | -5.82 | 0.425 |
|  | 45 | 82.6 ± 2.95 | 29.4 * | 0.000 |
|  | 50 | 93.8 ± 1.06 | 40.6 * | 0.000 |
|  | 55 | 91.9 ± 1.25 | 38.7 * | 0.000 |
| **WW2** | 25 | 8.66 ± 2.00 | N/A | N/A |
|  | 35 | 28.0 ± 3.13 | 19.3 * | 0.000 |
|  | 45 | 84.5 ± 3.50 | 75.9 * | 0.000 |
|  | 50 | 92.9 ± 1.54 | 84.2 * | 0.000 |
|  | 55 | 88.0 ± 1.48 | 79.4 * | 0.000 |
| **WW3** | 25 | 13.7 ± 3.17 | N/A | N/A |
|  | 35 | 32.7 ± 4.35 | 19.0 * | 0.000 |
|  | 45 | 81.3 ± 2.73 | 67.6 * | 0.000 |
|  | 50 | 93.2 ± 0.85 | 79.6 * | 0.000 |
|  | 55 | 93.3 ± 0.88 | 79.7 * | 0.000 |
| **WW4** | 25 | 19.2 ± 2.13 | N/A | N/A |
|  | 35 | 46.7 ± 3.12 | 27.5 * | 0.000 |
|  | 45 | 70.3 ± 3.90 | 51.1 * | 0.000 |
|  | 50 | 93.0 ± 1.27 | 73.8 * | 0.000 |
|  | 55 | 93.0 ± 1.09 | 73.8 * | 0.000 |

Supplementary Table 3 - Effect of salt stress on PCD levels in spring (SW) and winter (WW) wheat varieties. One-way ANOVA (Dunnett post-hoc) was used for statistical analysis which treated the SDW dataset as a control and compared all other group datasets against it. (*) indicates the mean difference is significant at the 0.05 level. Error bars = standard error of n ≥ 12 replicates.

| **Wheat Variety** | **NaCl (mM)** | **% PCD** | **Mean difference from SDW dataset (%)** | ***p*-value** |
| --- | --- | --- | --- | --- |
| **SW1** | SDW | 15.53 ± 1.73 | N/A | N/A |
|  | 50 | 20.44 ± 2.44 | 4.91 | 0.844 |
|  | 100 | 20.12 ± 2.07 | 4.59 | 0.896 |
|  | 150 | 22.52 ± 3.33 | 6.99 | 0.610 |
|  | 200 | 63.77 ± 5.49 | 48.3^*^ | 0.000 |
|  | 250 | 85.27 ± 4.32 | 69.8^*^ | 0.000 |
| **SW2** | SDW | 12.31 ± 1.61 | N/A | N/A |
|  | 50 | 15.01 ± 1.27 | 2.70 | 0.995 |
|  | 100 | 29.36 ± 5.10 | 17.0 | 0.054 |
|  | 150 | 21.49 ± 2.56 | 9.17 | 0.527 |
|  | 200 | 67.61 ± 6.57 | 55.3^*^ | 0.000 |
|  | 250 | 90.71 ± 3.14 | 78.4^*^ | 0.000 |
| **SW3** | SDW | 15.64 ± 1.71 | N/A | N/A |
|  | 50 | 26.00 ± 2.37 | 10.4^*^ | 0.048 |
|  | 100 | 24.57 ± 2.18 | 8.93 | 0.128 |
|  | 150 | 28.50 ± 1.56 | 12.9^*^ | 0.009 |
|  | 200 | 80.10 ± 4.80 | 64.5^*^ | 0.000 |
|  | 250 | 86.15 ± 2.96 | 70.5^*^ | 0.000 |
| **SW4** | SDW | 36.69 ± 4.07 | N/A | N/A |
|  | 50 | 34.44 ± 3.78 | -2.25 | 0.997 |
|  | 100 | 35.37 ± 3.20 | -1.31 | 1.000 |
|  | 150 | 62.56 ± 5.78 | 25.9^*^ | 0.000 |
|  | 200 | 76.45 ± 5.03 | 39.78^*^ | 0.000 |
|  | 250 | 91.57 ± 2.03 | 54.9^*^ | 0.000 |
| **WW1** | SDW | 26.89 ± 2.26 | N/A | N/A |
|  | 50 | 28.96 ± 2.94 | 2.07 | 0.995 |
|  | 100 | 32.70 ± 2.11 | 5.81 | 0.642 |
|  | 150 | 44.27 ± 4.27 | 17.4^*^ | 0.007 |
|  | 200 | 71.09 ± 4.89 | 44.2^*^ | 0.000 |
|  | 250 | 89.96 ± 2.58 | 63.1^*^ | 0.000 |
| **WW2** | SDW | 25.55 ± 1.92 | N/A | N/A |
|  | 50 | 26.91 ± 1.98 | 1.36 | 0.998 |
|  | 100 | 30.18 ± 3.04 | 4.63 | 0.773 |
|  | 150 | 37.32 ± 4.66 | 11.8^*^ | 0.037 |
|  | 200 | 87.44 ± 2.89 | 61.9^*^ | 0.000 |
|  | 250 | 81.66 ± 3.21 | 56.1^*^ | 0.000 |
| **WW3** | SDW | 18.01 ± 2.23 | N/A | N/A |
|  | 50 | 14.99 ± 2.64 | -3.01 | 0.966 |
|  | 100 | 16.81 ± 2.43 | -1.20 | 1.000 |
|  | 150 | 29.39 ± 4.54 | 11.4 | 0.101 |
|  | 200 | 80.49 ± 5.85 | 62.5^*^ | 0.000 |
|  | 250 | 91.04 ± 1.26 | 73.0^*^ | 0.000 |
| **WW4** | SDW | 11.25 ± 2.42 | N/A | N/A |
|  | 50 | 15.87 ± 2.31 | 4.62 | 0.649 |
|  | 100 | 18.49 ± 2.29 | 7.24 | 0.219 |
|  | 150 | 29.67 ± 4.32 | 18.4^*^ | 0.000 |
|  | 200 | 89.74 ± 2.60 | 78.5^*^ | 0.000 |
|  | 250 | 93.02 ± 2.16 | 81.8^*^ | 0.000 |

Supplementary Table 4 - Examining how single, combined and multiple individual stress exposures affects stress-induced PCD in wheat varieties. The initial stress cue (35 °C heat or 150 mM NaCl) is applied at the 0-min mark, followed by the second stress application at different time-points (30, 60 and 120-min). (A) H+S refers to heat stress as the initial cue, followed by salt stress, while (B) S+H refers to salt stress as the first cue, followed by heat stress at the relevant time-points. One-way ANOVA (Dunnett post-hoc) was used for statistical analysis which treated the single-stress dataset as a control and compared all other group datasets against it. (*) indicates the mean difference is significant at the 0.05 level. Error bars = standard error of n ≥ 4 replicates.

1. **H+S datasets**

| **Wheat Variety** | **Temperature (°C)** | **% PCD** | **Mean difference from H-only control (%)** | ***p*-value** |
| --- | --- | --- | --- | --- |
| **SW1** | H-only | 77.8 ± 2.69 | N/A | N/A |
|  | 0 | 74.2 ± 2.47 | 3.07 | 0.830 |
|  | 30 | 78.1 ± 2.61 | -1.00 | 0.997 |
|  | 60 | 72.8 ± 3.72 | 4.27 | 0.658 |
|  | 120 | 90.6 ± 2.68 | -11.80 | 0.133 |
| **SW2** | H-only | 21.3 ± 1.24 | N/A | N/A |
|  | 0 | 25.7 ± 1.79 | 4.40 | 0.982 |
|  | 30 | 28.5 ± 7.54 | 7.18 | 0.906 |
|  | 60 | 15.0 ± 2.53 | -6.30 | 0.938 |
|  | 120 | 38.2 ± 13.41 | 16.86 | 0.321 |
| **SW3** | H-only | 65.2 ± 4.90 | N/A | N/A |
|  | 0 | 44.8 ± 5.09 | 19.8^*^ | 0.013 |
|  | 30 | 56.3 ± 4.06 | 6.21 | 0.731 |
|  | 60 | 59.0 ± 5.29 | 6.45 | 0.729 |
|  | 120 | 73.8 ± 2.84 | -8.03 | 0.548 |
| **SW4** | H-only | 48.4 ± 5.54 | N/A | N/A |
|  | 0 | 46.6 ± 4.70 | 0.38 | 1.000 |
|  | 30 | 66.8 ± 2.95 | -20.2* | 0.032 |
|  | 60 | 66.1 ± 4.08 | -16.92 | 0.101 |
|  | 120 | 48.4 ± 9.42 | 0.62 | 1.000 |
| **WW1** | H-only | 51.1 ± 2.36 | N/A | N/A |
|  | 0 | 55.4 ± 4.60 | -4.49 | 0.728 |
|  | 30 | 63.0 ± 3.02 | -12.3^*^ | 0.042 |
|  | 60 | 73.0 ± 2.76 | -21.5^*^ | 0.000 |
|  | 120 | 75.1 ± 2.63 | -23.5^*^ | 0.000 |
| **WW2** | H-only | 65.4 ± 3.04 | N/A | N/A |
|  | 0 | 63.0 ± 2.35 | 1.02 | 0.998 |
|  | 30 | 69.9 ± 3.54 | -5.45 | 0.532 |
|  | 60 | 59.7 ± 2.07 | 5.60 | 0.508 |
|  | 120 | 58.4 ± 4.19 | 6.18 | 0.422 |
| **WW3** | H-only | 71.7 ± 4.77 | N/A | N/A |
|  | 0 | 59.8 ± 3.97 | 11.19 | 0.268 |
|  | 30 | 58.2 ± 5.16 | 9.08 | 0.445 |
|  | 60 | 61.2 ± 3.67 | 9.38 | 0.408 |
|  | 120 | 63.2 ± 6.23 | 5.74 | 0.796 |
| **WW4** | H-only | 63.5 ± 2.23 | N/A | N/A |
|  | 0 | 45.8 ± 4.39 | 18.33 | 0.077 |
|  | 30 | 49.3 ± 7.64 | 14.50 | 0.176 |
|  | 60 | 53.1 ± 6.15 | 12.09 | 0.360 |
|  | 120 | 64.2 ± 3.52 | -1.42 | 0.999 |

1. **S+H datasets**

| **Wheat Variety** | **Temperature (°C)** | **% PCD** | **Mean difference from S-only control (%)** | ***p*-value** |
| --- | --- | --- | --- | --- |
| **SW1** | S-only | 54.4 ± 6.26 | N/A | N/A |
|  | 0 | 62.3 ± 4.01 | -8.58 | 0.549 |
|  | 30 | 53.6 ± 5.61 | -1.29 | 1.000 |
|  | 60 | 82.1 ± 4.11 | -27.6^*^ | 0.025 |
|  | 120 | 77.3 ± 2.71 | -23.87 | 0.064 |
| **SW2** | S-only | 39.1 ± 5.44 | N/A | N/A |
|  | 0 | 38.0 ± 4.05 | -1.11 | 0.999 |
|  | 30 | 28.9 ± 5.57 | -10.23 | 0.356 |
|  | 60 | 16.0 ± 3.44 | -23.1^*^ | 0.004 |
|  | 120 | 29.6 ± 4.30 | -9.50 | 0.396 |
| **SW3** | S-only | 58.0 ± 4.23 | N/A | N/A |
|  | 0 | 44.4 ± 5.00 | 12.14 | 0.171 |
|  | 30 | 58.9 ± 4.76 | -2.01 | 0.992 |
|  | 60 | 56.2 ± 5.69 | 1.85 | 0.996 |
|  | 120 | 60.2 ± 3.57 | -1.25 | 0.999 |
| **SW4** | S-only | 37.7 ± 5.56 | N/A | N/A |
|  | 0 | 31.4 ± 4.06 | 4.30 | 0.938 |
|  | 30 | 55.3 ± 5.85 | -20.2^*^ | 0.032 |
|  | 60 | 56.9 ± 4.30 | -21.9^*^ | 0.013 |
|  | 120 | 48.6 ± 6.43 | -11.37 | 0.365 |
| **WW1** | S-only | 32.0 ± 6.80 | N/A | N/A |
|  | 0 | 49.5 ± 4.27 | -17.80404^*^ | 0.017 |
|  | 30 | 56.5 ± 2.34 | -24.60167^*^ | 0.001 |
|  | 60 | 65.4 ± 3.46 | -33.43500^*^ | 0.000 |
|  | 120 | 72.4 ± 2.91 | -40.46865^*^ | 0.000 |
| **WW2** | S-only | 50.0 ± 3.91 | N/A | N/A |
|  | 0 | 44.0 ± 6.58 | 5.51 | 0.750 |
|  | 30 | 54.5 ± 3.28 | -4.38 | 0.866 |
|  | 60 | 62.0 ± 1.98 | -11.72 | 0.167 |
|  | 120 | 69.6 ± 2.42 | -20.6^*^ | 0.003 |
| **WW3** | S-only | 66.9 ± 4.21 | N/A | N/A |
|  | 0 | 54.9 ± 5.19 | 13.36 | 0.060 |
|  | 30 | 50.9 ± 3.31 | 16.5^*^ | 0.013 |
|  | 60 | 71.2 ± 3.72 | -2.32 | 0.981 |
|  | 120 | 66.0 ± 3.52 | 3.31 | 0.936 |
| **WW4** | S-only | 59.2 ± 4.16 | N/A | N/A |
|  | 0 | 55.7 ± 4.96 | 1.51 | 0.999 |
|  | 30 | 59.9 ± 3.03 | -1.87 | 0.998 |
|  | 60 | 51.8 ± 6.78 | 6.27 | 0.836 |
|  | 120 | 52.0 ± 6.93 | 6.53 | 0.844 |

Supplementary Table 5 - Overall trends noted in stressed wheat seedlings by varying the initial stress cue. One-way ANOVA (Dunnett post-hoc) was used for statistical analysis which treated the single-stress dataset as a control and compared all other group datasets against it. (*) indicates the mean difference is significant at the 0.05 level. Values represent the average PCD levels across the eight varieties. Error bars = standard error of n ≥ 4 replicates.

| **Wheat Variety** | **Temperature (°C)** | **% PCD** | **Mean difference from single stress control (%)** | ***p*-value** |
| --- | --- | --- | --- | --- |
| **H+S** | H-only | 34.1 ± 1.65 | N/A | N/A |
|  | 0 | 40.8 ± 2.60 | 6.63^*^ | 0.038 |
|  | 30 | 34.1 ± 2.60 | -0.06 | 1.000 |
|  | 60 | 33.7 ± 2.64 | -0.44 | 0.999 |
|  | 120 | 33.3 ± 2.70 | -0.86 | 0.993 |
| **S+H** | S-only | 46.3 ± 2.16 | N/A | N/A |
|  | 0 | 47.8 ± 2.63 | 1.50 | 0.946 |
|  | 30 | 40.6 ± 2.68 | -5.69 | 0.110 |
|  | 60 | 34.7 ± 2.69 | -11.6^*^ | 0.000 |
|  | 120 | 35.5 ± 2.69 | -10.8^*^ | 0.000 |

Supplementary Table 6 - Independent samples *t*-test of individual varieties examining induced tolerance across individual wheat varieties and different initial stress cues: (A) H+S and (B) S+H. Induced tolerance values represent the merged PCD levels across 30, 60 and 120-min datasets, where error bars = standard error of n ≥ 4 replicates. (*) indicates the mean difference is significant at the 0.05 level.

1. **H+S datasets**

| **SW1** | **(H+S)** |  |  |  |  |
| --- | --- | --- | --- | --- | --- |
|  | **Group Statistics** | | ***t*-test for Equality of Means** | | |
| Stress type | Mean | Std. Error Mean | Sig. (2-tailed) | Mean Difference | Std. Error Difference |
| Single stress | 21.0 | 2.58 | 0.926 | 0.31 | 3.30 |
| Induced tolerance | 20.7 | 2.07 |  |  |  |
|  |  |  |  |  |  |
| **SW2** | **(H+S)** |  |  |  |  |
|  | **Group Statistics** | | ***t*-test for Equality of Means** | | |
| Stress type | Mean | Std. Error Mean | Sig. (2-tailed) | Mean Difference | Std. Error Difference |
| Single stress | 21.3 | 1.24 | 0.278 | -6.35 | 5.73 |
| Induced tolerance | 27.7 | 5.59 |  |  |  |
|  |  |  |  |  |  |
| **SW3** | **(H+S)** |  |  |  |  |
|  | **Group Statistics** | | ***t*-test for Equality of Means** | | |
| Stress type | Mean | Std. Error Mean | Sig. (2-tailed) | Mean Difference | Std. Error Difference |
| Single stress | 33.2 | 4.90 | 0.785 | -1.54 | 5.57 |
| Induced tolerance | 34.7 | 2.65 |  |  |  |
|  |  |  |  |  |  |
| **SW4** | **(H+S)** |  |  |  |  |
|  | **Group Statistics** | | ***t*-test for Equality of Means** | | |
| Stress type | Mean | Std. Error Mean | Sig. (2-tailed) | Mean Difference | Std. Error Difference |
| Single stress | 49.5 | 5.54 | 0.064 | 12.71 | 6.57 |
| Induced tolerance | 36.8 | 3.53 |  |  |  |
|  |  |  |  |  |  |
| **WW1** | **(H+S)** |  |  |  |  |
|  | **Group Statistics** | | ***t*-test for Equality of Means** | | |
| Stress type | Mean | Std. Error Mean | Sig. (2-tailed) | Mean Difference | Std. Error Difference |
| Single stress | 47.5 | 2.39 | 0.000* | 19.10 | 2.98 |
| Induced tolerance | 28.4 | 1.78 |  |  |  |
|  |  |  |  |  |  |
| **WW2** | **(H+S)** |  |  |  |  |
|  | **Group Statistics** | | ***t*-test for Equality of Means** | | |
| Stress type | Mean | Std. Error Mean | Sig. (2-tailed) | Mean Difference | Std. Error Difference |
| Single stress | 34.2 | 3.04 | 0.570 | -2.11 | 3.65 |
| Induced tolerance | 36.3 | 2.02 |  |  |  |
|  |  |  |  |  |  |
| **WW3** | **(H+S)** |  |  |  |  |
|  | **Group Statistics** | | ***t*-test for Equality of Means** | | |
| Stress type | Mean | Std. Error Mean | Sig. (2-tailed) | Mean Difference | Std. Error Difference |
| Single stress | 27.0 | 4.77 | 0.155 | -8.21 | 5.38 |
| Induced tolerance | 35.2 | 2.49 |  |  |  |
|  |  |  |  |  |  |
| **WW4** | **(H+S)** |  |  |  |  |
|  | **Group Statistics** | | ***t*-test for Equality of Means** | | |
| Stress type | Mean | Std. Error Mean | Sig. (2-tailed) | Mean Difference | Std. Error Difference |
| Single stress | 34.3 | 2.12 | 0.051 | -8.59 | 4.31 |
| Induced tolerance | 42.9 | 3.75 |  |  |  |

1. **S+H datasets**

| **SW1** | **S+H** |  |  |  |  |
| --- | --- | --- | --- | --- | --- |
|  | **Group Statistics** | | ***t*-test for Equality of Means** | | |
| Stress type | Mean | Std. Error Mean | Sig. (2-tailed) | Mean Difference | Std. Error Difference |
| Single stress | 44.5 | 6.13 | 0.086 | 13.52 | 7.53 |
| Induced tolerance | 31.0 | 4.38 |  |  |  |
|  |  |  |  |  |  |
| **SW2** | **S+H** |  |  |  |  |
|  | **Group Statistics** | | ***t*-test for Equality of Means** | | |
| Stress type | Mean | Std. Error Mean | Sig. (2-tailed) | Mean Difference | Std. Error Difference |
| Single stress | 39.1 | 5.44 | 0.038* | 14.44 | 6.11 |
| Induced tolerance | 24.7 | 2.76 |  |  |  |
|  |  |  |  |  |  |
| **SW3** | **S+H** |  |  |  |  |
|  | **Group Statistics** | | ***t*-test for Equality of Means** | | |
| Stress type | Mean | Std. Error Mean | Sig. (2-tailed) | Mean Difference | Std. Error Difference |
| Single stress | 39.9 | 4.23 | 0.894 | 0.66 | 4.92 |
| Induced tolerance | 39.2 | 2.52 |  |  |  |
|  |  |  |  |  |  |
| **SW4** | **S+H** |  |  |  |  |
|  | **Group Statistics** | | ***t*-test for Equality of Means** | | |
| Stress type | Mean | Std. Error Mean | Sig. (2-tailed) | Mean Difference | Std. Error Difference |
| Single stress | 60.9 | 5.56 | 0.010* | 18.03 | 6.35 |
| Induced tolerance | 42.9 | 3.07 |  |  |  |
|  |  |  |  |  |  |
| **WW1** | **S+H** |  |  |  |  |
|  | **Group Statistics** | | ***t*-test for Equality of Means** | | |
| Stress type | Mean | Std. Error Mean | Sig. (2-tailed) | Mean Difference | Std. Error Difference |
| Single stress | 67.4 | 6.96 | 0.001* | 33.04 | 7.24 |
| Induced tolerance | 34.4 | 2.01 |  |  |  |
|  |  |  |  |  |  |
| **WW2** | **S+H** |  |  |  |  |
|  | **Group Statistics** | | ***t*-test for Equality of Means** | | |
| Stress type | Mean | Std. Error Mean | Sig. (2-tailed) | Mean Difference | Std. Error Difference |
| Single stress | 48.8 | 3.91 | 0.012* | 12.24 | 4.32 |
| Induced tolerance | 36.6 | 1.83 |  |  |  |
|  |  |  |  |  |  |
| **WW3** | **S+H** |  |  |  |  |
|  | **Group Statistics** | | ***t*-test for Equality of Means** | | |
| Stress type | Mean | Std. Error Mean | Sig. (2-tailed) | Mean Difference | Std. Error Difference |
| Single stress | 29.0 | 4.21 | 0.216 | -6.11 | 4.78 |
| Induced tolerance | 35.1 | 2.27 |  |  |  |
|  |  |  |  |  |  |
| **WW4** | **S+H** |  |  |  |  |
|  | **Group Statistics** | | ***t*-test for Equality of Means** | | |
| Stress type | Mean | Std. Error Mean | Sig. (2-tailed) | Mean Difference | Std. Error Difference |
| Single stress | 39.4 | 4.30 | 0.476 | -4.03 | 5.58 |
| Induced tolerance | 43.5 | 3.55 |  |  |  |

Supplementary Table 7 – Independent samples *t*-test of individual varieties examining basal tolerance using combined stress. (A) H+S and (B) S+H. Combined stress reflects the PCD levels recorded after simultaneous stress exposure (H+S or S+H) at the 0-min mark. Error bars = standard error of n ≥4 replicates. (*) indicates the mean difference is significant at the 0.05 level.

1. **H+S datasets**

| **SW1** | **(H+S)** |  |  |  |  |
| --- | --- | --- | --- | --- | --- |
|  | **Group Statistics** | | ***t*-test for Equality of Means** | | |
| Stress type | Mean | Std. Error Mean | Sig. (2-tailed) | Mean Difference | Std. Error Difference |
| Single stress | 21.0 | 2.58 | 0.389 | -3.1 | 3.50 |
| Combined stress | 24.0 | 2.37 |  |  |  |
|  |  |  |  |  |  |
| **SW2** | **(H+S)** |  |  |  |  |
|  | **Group Statistics** | | ***t*-test for Equality of Means** | | |
| Stress type | Mean | Std. Error Mean | Sig. (2-tailed) | Mean Difference | Std. Error Difference |
| Single stress | 21.3 | 1.24 | 0.066 | -4.4 | 2.18 |
| Combined stress | 25.7 | 1.79 |  |  |  |
|  |  |  |  |  |  |
| **SW3** | **(H+S)** |  |  |  |  |
|  | **Group Statistics** | | ***t*-test for Equality of Means** | | |
| Stress type | Mean | Std. Error Mean | Sig. (2-tailed) | Mean Difference | Std. Error Difference |
| Single stress | 33.2 | 4.90 | 0.009* | -19.8 | 7.04 |
| Combined stress | 53.0 | 5.05 |  |  |  |
|  |  |  |  |  |  |
| **SW4** | **(H+S)** |  |  |  |  |
|  | **Group Statistics** | | ***t*-test for Equality of Means** | | |
| Stress type | Mean | Std. Error Mean | Sig. (2-tailed) | Mean Difference | Std. Error Difference |
| Single stress | 49.5 | 5.54 | 0.959 | -0.4 | 7.29 |
| Combined stress | 49.9 | 4.73 |  |  |  |
|  |  |  |  |  |  |
| **WW1** | **(H+S)** |  |  |  |  |
|  | **Group Statistics** | | ***t*-test for Equality of Means** | | |
| Stress type | Mean | Std. Error Mean | Sig. (2-tailed) | Mean Difference | Std. Error Difference |
| Single stress | 43.0 | 4.65 | 0.401 | -4.5 | 5.23 |
| Combined stress | 47.5 | 2.39 |  |  |  |
|  |  |  |  |  |  |
| **WW2** | **(H+S)** |  |  |  |  |
|  | **Group Statistics** | | ***t*-test for Equality of Means** | | |
| Stress type | Mean | Std. Error Mean | Sig. (2-tailed) | Mean Difference | Std. Error Difference |
| Single stress | 35.2 | 2.54 | 0.800 | 1.0 | 3.96 |
| Combined stress | 34.2 | 3.04 |  |  |  |
|  |  |  |  |  |  |
| **WW3** | **(H+S)** |  |  |  |  |
|  | **Group Statistics** | | ***t*-test for Equality of Means** | | |
| Stress type | Mean | Std. Error Mean | Sig. (2-tailed) | Mean Difference | Std. Error Difference |
| Single stress | 38.2 | 3.76 | 0.085 | 11.2 | 6.08 |
| Combined stress | 27.0 | 4.77 |  |  |  |
|  |  |  |  |  |  |
| **WW4** | **(H+S)** |  |  |  |  |
|  | **Group Statistics** | | ***t*-test for Equality of Means** | | |
| Stress type | Mean | Std. Error Mean | Sig. (2-tailed) | Mean Difference | Std. Error Difference |
| Single stress | 52.6 | 4.57 | 0.002* | 18.3 | 5.04 |
| Combined stress | 34.3 | 2.12 |  |  |  |

1. **S+H datasets**

| **SW1** | **(S+H)** |  |  |  |  |
| --- | --- | --- | --- | --- | --- |
|  | **Group Statistics** | | ***t*-test for Equality of Means** | | |
| Stress type | Mean | Std. Error Mean | Sig. (2-tailed) | Mean Difference | Std. Error Difference |
| Single stress | 35.9 | 3.95 | 0.253 | -8.6 | 7.29 |
| Combined stress | 44.5 | 6.13 |  |  |  |
|  |  |  |  |  |  |
| **SW2** | **(S+H)** |  |  |  |  |
|  | **Group Statistics** | | ***t*-test for Equality of Means** | | |
| Stress type | Mean | Std. Error Mean | Sig. (2-tailed) | Mean Difference | Std. Error Difference |
| Single stress | 38.0 | 4.05 | 0.873 | -1.1 | 6.78 |
| Combined stress | 39.1 | 5.44 |  |  |  |
|  |  |  |  |  |  |
| **SW3** | **(S+H)** |  |  |  |  |
|  | **Group Statistics** | | ***t*-test for Equality of Means** | | |
| Stress type | Mean | Std. Error Mean | Sig. (2-tailed) | Mean Difference | Std. Error Difference |
| Single stress | 52.1 | 4.59 | 0.064 | 12.1 | 6.24 |
| Combined stress | 39.9 | 4.23 |  |  |  |
|  |  |  |  |  |  |
| **SW4** | **(S+H)** |  |  |  |  |
|  | **Group Statistics** | | ***t*-test for Equality of Means** | | |
| Stress type | Mean | Std. Error Mean | Sig. (2-tailed) | Mean Difference | Std. Error Difference |
| Single stress | 65.2 | 4.49 | 0.553 | 4.3 | 7.14 |
| Combined stress | 60.9 | 5.56 |  |  |  |
|  |  |  |  |  |  |
| **WW1** | **(S+H)** |  |  |  |  |
|  | **Group Statistics** | | ***t*-test for Equality of Means** | | |
| Stress type | Mean | Std. Error Mean | Sig. (2-tailed) | Mean Difference | Std. Error Difference |
| Single stress | 49.6 | 4.40 | 0.044* | -17.8 | 8.23 |
| Combined stress | 67.4 | 6.96 |  |  |  |
|  |  |  |  |  |  |
| **WW2** | **(S+H)** |  |  |  |  |
|  | **Group Statistics** | | ***t*-test for Equality of Means** | | |
| Stress type | Mean | Std. Error Mean | Sig. (2-tailed) | Mean Difference | Std. Error Difference |
| Single stress | 54.3 | 6.91 | 0.496 | 5.5 | 7.94 |
| Combined stress | 48.8 | 3.91 |  |  |  |
|  |  |  |  |  |  |
| **WW3** | **(S+H)** |  |  |  |  |
|  | **Group Statistics** | | ***t*-test for Equality of Means** | | |
| Stress type | Mean | Std. Error Mean | Sig. (2-tailed) | Mean Difference | Std. Error Difference |
| Single stress | 42.4 | 4.90 | 0.049* | 13.4 | 6.46 |
| Combined stress | 29.0 | 4.21 |  |  |  |
|  |  |  |  |  |  |
| **WW4** | **(S+H)** |  |  |  |  |
|  | **Group Statistics** | | ***t*-test for Equality of Means** | | |
| Stress type | Mean | Std. Error Mean | Sig. (2-tailed) | Mean Difference | Std. Error Difference |
| Single stress | 41.0 | 4.83 | 0.818 | 1.5 | 6.47 |
| Combined stress | 39.4 | 4.30 |  |  |  |

Supplementary Table 8 - Independent samples *t-*test of individual varieties examining the effects of applying different stress cues as the initial cue on PCD levels. Inputted data consisted of PCD levels scored across 0, 30, 60 and 120-mins. (*) indicates the mean difference is significant at the 0.05 level.

| **SW1** |  |  |  |  |  |
| --- | --- | --- | --- | --- | --- |
|  | **Group Statistics** | | ***t*-test for Equality of Means** | | |
| Initial Stress cue | Mean | Std. Error Mean | Sig. (2-tailed) | Mean Difference | Std. Error Difference |
| H+S | 21.8 | 1.59 | 0.002* | -11.4 | 3.4 |
| S+H | 33.2 | 2.98 |  |  |  |
|  |  |  |  |  |  |
| **SW2** |  |  |  |  |  |
|  | **Group Statistics** | | ***t*-test for Equality of Means** | | |
| Initial Stress cue | Mean | Std. Error Mean | Sig. (2-tailed) | Mean Difference | Std. Error Difference |
| H+S | 27.2 | 4.24 | 0.856 | -0.9 | 4.9 |
| S+H | 28.1 | 2.48 |  |  |  |
|  |  |  |  |  |  |
| **SW3** |  |  |  |  |  |
|  | **Group Statistics** | | ***t*-test for Equality of Means** | | |
| Initial Stress cue | Mean | Std. Error Mean | Sig. (2-tailed) | Mean Difference | Std. Error Difference |
| H+S | 39.3 | 2.56 | 0.362 | -3.2 | 3.5 |
| S+H | 42.4 | 2.33 |  |  |  |
|  |  |  |  |  |  |
| **SW4** |  |  |  |  |  |
|  | **Group Statistics** | | ***t*-test for Equality of Means** | | |
| Initial Stress cue | Mean | Std. Error Mean | Sig. (2-tailed) | Mean Difference | Std. Error Difference |
| H+S | 40.1 | 2.98 | 0.042* | -8.5 | 4.1 |
| S+H | 48.6 | 2.86 |  |  |  |
|  |  |  |  |  |  |
| **WW1** |  |  |  |  |  |
|  |  | | ***t*-test for Equality of Means** | | |
| Initial Stress cue | Mean | Std. Error Mean | Sig. (2-tailed) | Mean Difference | Std. Error Difference |
| H+S | 32.5 | 2.03 | 0.046* | -5.9 | 2.9 |
| S+H | 38.4 | 2.08 |  |  |  |
|  |  |  |  |  |  |
|  |  |  |  |  |  |
|  |  |  |  |  |  |
| **WW2** |  |  |  |  |  |
|  | **Group Statistics** | | ***t*-test for Equality of Means** | | |
| Initial Stress cue | Mean | Std. Error Mean | Sig. (2-tailed) | Mean Difference | Std. Error Difference |
| H+S | 36 | 1.63 | 0.088 | -5.1 | 2.9 |
| S+H | 41.1 | 2.44 |  |  |  |
|  |  |  |  |  |  |
| **WW3** |  |  |  |  |  |
|  | **Group Statistics** | | ***t*-test for Equality of Means** | | |
| Initial Stress cue | Mean | Std. Error Mean | Sig. (2-tailed) | Mean Difference | Std. Error Difference |
| H+S | 36 | 2.07 | 0.75 | -0.9 | 3 |
| S+H | 37 | 2.13 |  |  |  |
|  |  |  |  |  |  |
| **WW4** |  |  |  |  |  |
|  | **Group Statistics** | | ***t*-test for Equality of Means** | | |
| Initial Stress cue | Mean | Std. Error Mean | Sig. (2-tailed) | Mean Difference | Std. Error Difference |
| H+S | 45.1 | 3.1 | 0.603 | 2.2 | 4.3 |
| S+H | 42.9 | 2.94 |  |  |  |


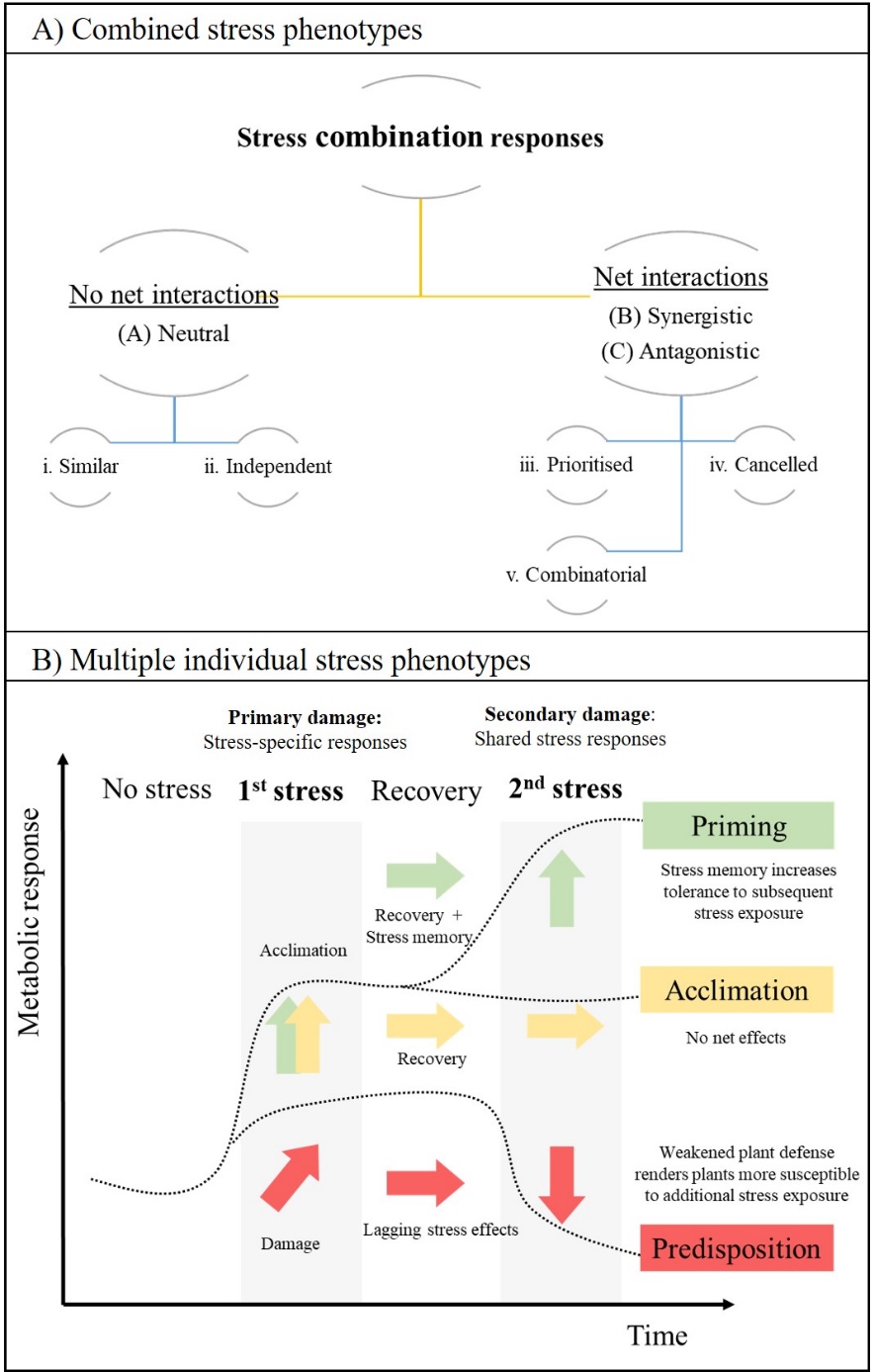


Supplementary Figure 1 - (A) The stress response phenotype after combined stress can be grouped into three categories: neutral, synergistic and antagonistic (Mittler, 2006), which can be further sub-divided into five sub-categories: similar, independent, prioritized, cancelled and combinatorial (Rasmussen et al., 2013). (B) The putative positive, neutral and negative stress response phenotype of plants under multiple individual stress applications. The first non-lethal stress cue either primes (positive and neutral) or predisposes (negative) plants to subsequent stress encounters. Image adapted with permission from Walter et al., (2013).


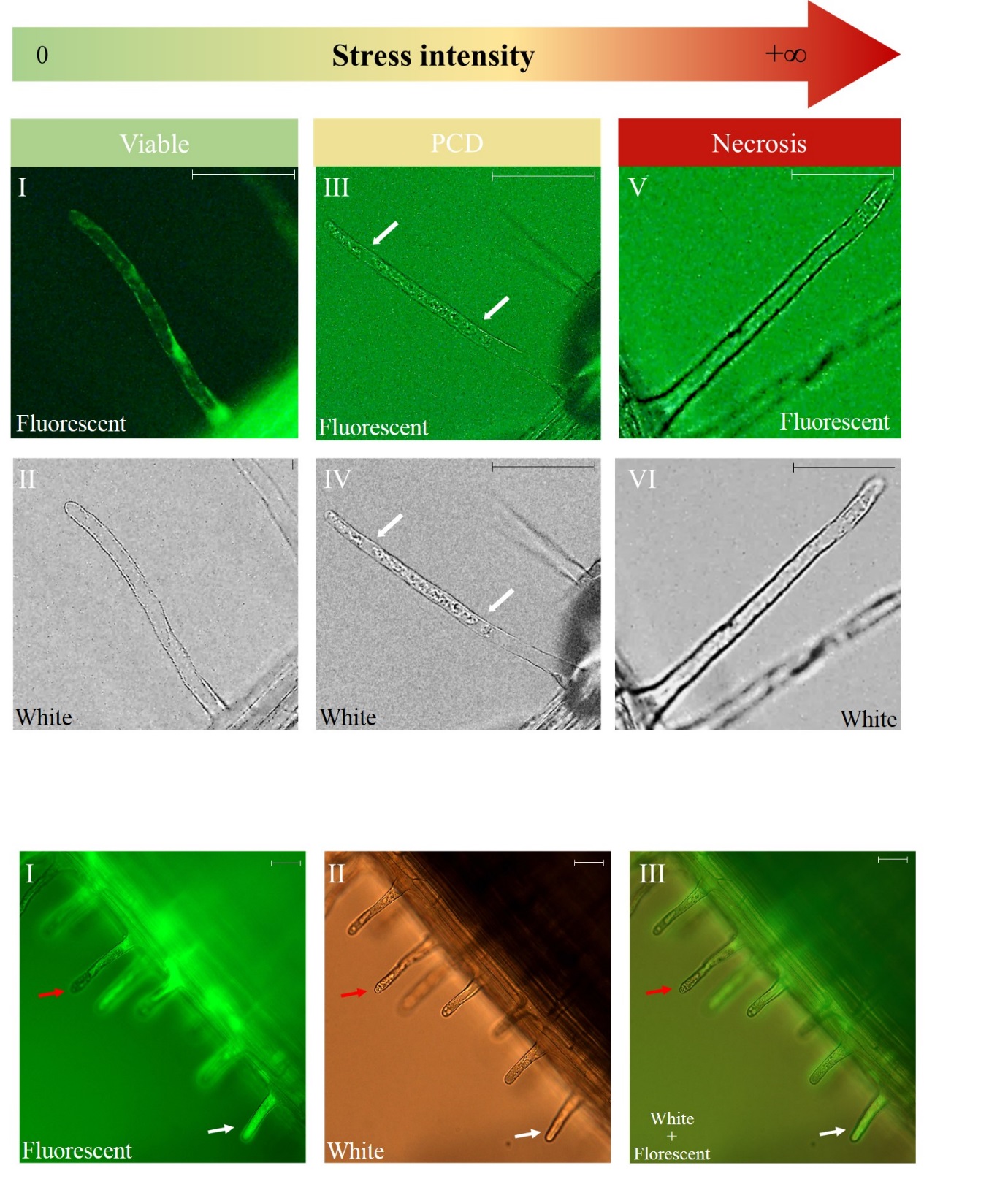


Supplementary Figure 2 - (A) Cell morphology of FDA-stained root hairs of the model organism *A. thaliana* heat-shocked for 10 minutes and viewed under fluorescent (I, III and V) and white light (II, IV and VI) 24 hours later. Viable root hairs are FDA positive and exhibit fluorescence (I and II – no heat shock), PCD root hairs are FDA negative and have a retracted cytoplasm indicated by white arrows (III and IV – 50 °C heat shock), and necrotic root hairs are FDA negative but do not have a retracted cytoplasm (V and VI – 80 °C heat shock). Scale bars: I-VI 10 µm. (B) Mixed markers (FDA positive but retracted cytoplasm) of 150 mM NaCl salt-shocked wheat (*T. aestivum*) root hairs under fluorescent light (I), full spectrum white light (II), and when both images are superimposed (III). Note the difference in fluorescence magnitude between viable root hairs (white arrow), and PCD root hairs (red arrow). Images captured using an Olympus BX61 microscope. Scale bars: I-III 10 µm.


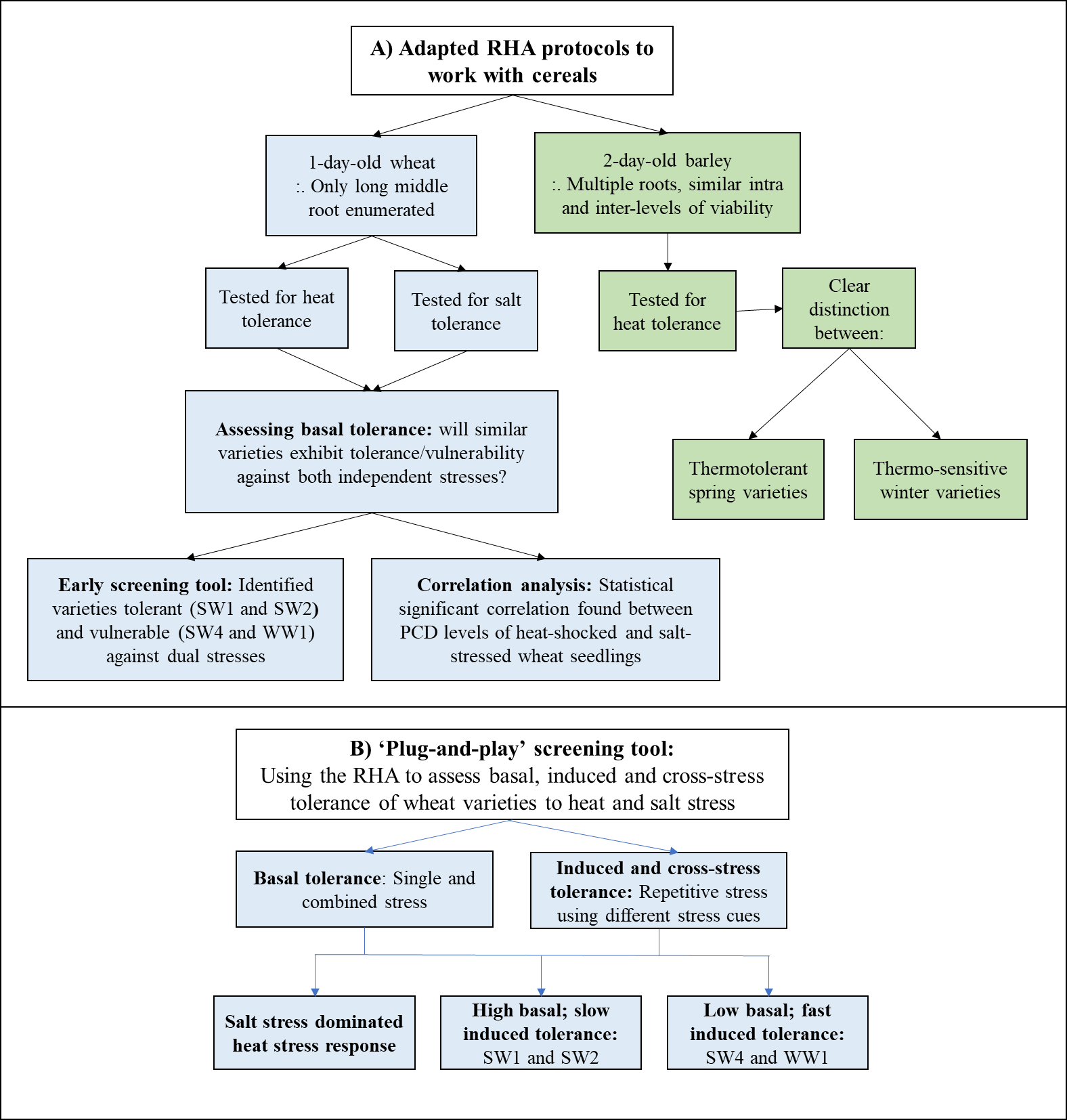


Supplementary Figure 3 – Summary of findings obtained in the study. (A) Adaptation of the RHA to work in cereals by establishing heat and/or salt-stress response curves in wheat and barley seedlings. (B) Assessing the basal, induced and cross-stress tolerance of wheat varieties against heat and salt stress,
